# AnchorFCI: harnessing genetic anchors for enhanced causal discovery of cardiometabolic disease pathways

**DOI:** 10.3389/fgene.2024.1436947

**Published:** 2024-12-09

**Authors:** Adèle H. Ribeiro, Milena Crnkovic, Jaqueline Lopes Pereira, Regina Mara Fisberg, Flavia Mori Sarti, Marcelo Macedo Rogero, Dominik Heider, Andressa Cerqueira

**Affiliations:** ^1^ Institute of Medical Informatics, University of Münster, Münster, Germany; ^2^ Department of Statistics, Federal University of São Carlos (UFSCar), São Carlos, Brazil; ^3^ Department of Nutrition, School of Public Health, University of São Paulo, São Paulo, Brazil; ^4^ School of Arts, Sciences and Humanities (EACH), University of São Paulo, São Paulo, Brazil

**Keywords:** causal discovery, explainability, RFCI, genetic anchors, unfaithfulness, partial ancestral graphs, causal effect identification, cardiometabolic risk factors

## Abstract

**Introduction:**

Cardiometabolic diseases, a major global health concern, stem from complex interactions of lifestyle, genetics, and biochemical markers. While extensive research has revealed strong associations between various risk factors and these diseases, latent confounding and limited causal discovery methods hinder understanding of their causal relationships, essential for mechanistic insights and developing effective prevention and intervention strategies.

**Methods:**

We introduce anchorFCI, a novel adaptation of the conservative Really Fast Causal Inference (RFCI) algorithm, designed to enhance robustness and discovery power in causal learning by strategically selecting and integrating reliable anchor variables from a set of variables known not to be caused by the variables of interest. This approach is well-suited for studies of phenotypic, clinical, and sociodemographic data, using genetic variables that are recognized to be unaffected by these factors. We demonstrate the method’s effectiveness through simulation studies and a comprehensive causal analysis of the 2015 ISA-Nutrition dataset, featuring both anchorFCI for causal discovery and state-of-the-art effect size identification tools from Judea Pearl’s framework, showcasing a robust, fully data-driven causal inference pipeline.

**Results:**

Our simulation studies reveal that anchorFCI effectively enhances robustness and discovery power while handles latent confounding by integrating reliable anchor variables and their non-ancestral relationships. The 2015 ISA-Nutrition dataset analysis not only supports many established causal relationships but also elucidates their interconnections, providing a clearer understanding of the complex dynamics and multifaceted nature of cardiometabolic risk.

**Discussion:**

AnchorFCI holds significant potential for reliable causal discovery in complex, multidimensional datasets. By effectively integrating non-ancestral knowledge and addressing latent confounding, it is well-suited for various applications requiring robust causal inference from observational studies, providing valuable insights in epidemiology, genetics, and public health.

## 1 Introduction

Cardiometabolic diseases, including cardiovascular disease and metabolic syndrome, are major global health concerns. They significantly contribute to the global burden of disease and mortality, impacting not only individual health but also societies, health systems, and economies on a global scale (Miranda et al., 2019; Rao, 2018). Numerous studies have identified a range of risk factors that contribute to the development and progression of cardiometabolic diseases. These factors arise from a complex interplay of lifestyle choices, genetic predispositions, and pre-existing conditions such as obesity, hypertension, inflammation, insulin resistance, type 2 diabetes, and dyslipidemia (Valenzuela et al., 2023; Kälsch et al., 2015; Wilson and Meigs, 2008).

Understanding the interconnections among these factors in a causal manner is essential for gaining insights into the mechanisms at play and accurately assessing an individual’s risk profile. This knowledge empowers clinicians to make informed decisions and aid researchers in the development of interventions and prevention strategies that are both effective and tailored to the specific needs of each patient.

Traditionally, causality has been established through experimental studies, following the Bradford-Hill considerations on causality (Höfler, 2005). However, conducting controlled experiments on humans presents significant challenges and ethical limitations, especially when addressing long-term effects and interventions that may necessitate lifestyle changes or carry health risks for participants. In this work, we aim to identify the causal relationships among various cardiometabolic risk factors observed in the Health Survey of São Paulo with a Focus on Nutrition Study (2015 ISA-Nutrition), a comprehensive epidemiological study conducted in São Paulo, Brazil (Fisberg et al., 2018). Observational studies are invaluable tools for gaining insights into the complexities of the real world, but they face critical challenges due to biases. Significant statistical associations among variables do not always indicate causality; they may, in fact, be entirely spurious, originating from confounding factors or other issues such as selection bias. This complexity emphasizes the need for rigorous methodologies to address biases in causal inference from observational data.

For our analysis, we consider Judea Pearl’s framework of causation (Pearl, 2000a). This framework represents a seminal advancement in data analysis, enabling us to move beyond mere correlations and discern causal relationships directly from observational data, thus reflecting the rigor typically associated with controlled experiments. Causality is articulated in an intuitive manner: a variable X is considered a cause of another variable Y if an intervention on X (e.g., setting it to a specific value, X=x ) results in an expected change in Y . Notably, certain causal discovery algorithms are capable of identifying, despite hidden confounding, the graphical structure of the class of causal models that may have generated the observed data (Zhang, 2008b). These algorithms, complemented by emerging tools for effect identification from their outputs (Maathuis and Colombo, 2015; Perkovi et al., 2018; Jaber et al., 2022), have paved the way for a powerful, data-driven approach to causal inference.

As our primary methodological contribution, we propose the anchor Fast Causal Inference (anchorFCI) algorithm, designed to robustly uncover causal relationships among a set of variables by leveraging an additional set comprising only non-ancestors (non-causes) of the variables of interest. This is achieved by strategically selecting reliable anchors and integrating knowledge of their non-ancestral relationships into the conservative Really Fast Causal Inference (RFCI) algorithm (Colombo et al., 2012), renowned for its effectiveness and robustness in scenarios with latent confounding and limited sample sizes. Specifically, anchorFCI identifies as reliable anchors those variables from the additional set that are significantly associated with the variables of interest and form unambiguous triplets during the conservative orientation phase of the algorithm. Then, it incorporates knowledge of their non-ancestral relationships as outlined in Section 2.4.2. AnchorFCI not only enforces the appropriate arrowheads but also utilizes an adapted skeleton phase that prevents conditional independence tests between anchor variables conditioned on non-anchor variables. This adaptation is vital for preserving the integrity of the encoding of the conditional independencies implied by the final graph while reducing the number of required conditional independence tests, thereby enhancing the algorithm’s scalability and robustness.

Our proposed approach is particularly well-suited for causal discovery among phenotypes, clinical factors, and sociodemographic variables when information on genetic variants, such as single nucleotide polymorphisms (SNPs), is available. This is because we can leverage the well-established understanding that genotypes are not influenced by any of the non-genetic variables. It is crucial to emphasize that, while this approach shares some similarities with Mendelian randomization principles (Davey Smith and Hemani, 2014; Ribeiro et al., 2016), it does not assume that SNPs identified as anchors are valid instruments (de Leeuw et al., 2022). Rather, the anchorFCI algorithm solely relies on conditional independence tests to identify genetic anchor variables and uncover causal relationships.

In analyzing the 2015 ISA-Nutrition dataset, our approach has successfully unveiled the causal connections among all examined phenotype and clinical variables, by leveraging SNPs identified in Genome-Wide Association Studies (GWAS). Furthermore, by employing effect identification tools, we have successfully identified and estimated the causal effects associated to all uncovered causal relationships. The results corroborate numerous well-established relationships, while also providing a deeper understanding of the intricate network of connections among various cardiometabolic risk factors. Additionally, they demonstrate the potential of robust data-driven causal inference methods in addressing complex and multifactorial diseases, paving the way for the development of more effective interventions and treatments.

## 2 Materials and methods

### 2.1 Study design and data collection

Our analysis focused on 681 individuals who were not genetically closely related, selected from a total of 841 participants in the *2015 Health Survey of São Paulo with a Focus on Nutrition Study* (2015 ISA-Nutrition) – a subset of the cross-sectional population-based *2015 Health Survey of São Paulo* (2015 ISA-Capital). The primary goal of the 2015 ISA-Nutrition study is to investigate the relationships between lifestyle choices, biochemical markers, and genetics in the development of cardiometabolic diseases among residents of São Paulo city, the largest Brazilian city located in the Southeastern region of the country. For more comprehensive information about the study, refer to Fisberg et al. (2018).

The survey targeted a probabilistic sample of individuals age ≥12 , residing in permanent households within the urban area of São Paulo city, excluding those who were pregnant or lactating. The sampling process involved two stages with stratification by clusters (urban census tracts and households) to ensure representative coverage at the population level. During the year of 2015, ISA-Capital collected sociodemographic data, information regarding the use of health services, lifestyle, and other relevant information through a structured questionnaire administered in the households by trained interviewers (Alves et al., 2018). Anthropometric data, blood pressure measurements, and blood samples were collected by trained nurses during a second visit to the participant’s household. Detailed protocols for these measurements are also available in Fisberg et al. (2018).

Genomic DNA was obtained from peripheral blood samples extracted by automated method. SNPs were assessed using the ${\text{Axiom}}^{\text{TM}}$ 2.0 Precision Medicine Research Array in the Thermo Fisher Scientific Laboratory (Affymetrix Inc., Santa Clara, CA).

This study has been conducted according to the principles expressed in the Declaration of Helsinki and was approved by the Ethics Committee on Research of the School of Public Health of the University of São Paulo (#30848914.7.0000.5421). All participants authorized their genotyping and signed a written informed consent/assent before entering the study and, if they were adolescents, also their proxies. The data and samples were anonymized after collection.

### 2.2 Sample selection and description of the variables

The 2015 ISA-Nutrition study incorporates genetic data from 841 individuals, with some being relatives. Notably, conducting causal analysis on samples from genetically or familial-related individuals necessitates careful consideration of the underlying dependence structure among them (Ribeiro and Soler, 2020). Since addressing this issue is beyond the scope of this study, we excluded individuals who might share a parental relationship, resulting to a final sample size of 681 individuals. Specifically, the genomic relationship matrix (GRM) was computed for all 841 individuals and our analysis was limited to individuals with a genetic distance of ≤0.125 , indicating relationships beyond second-degree relatives.

The dataset comprises a diverse array of sociodemographic, clinical, and genetic data. Additionally, it includes principal components of global ancestry allowing for adjustment for population stratification effects. This adjustment is particularly crucial in highly admixed populations such as the Brazilian population. The principal components of global ancestry are estimated using a larger set of SNPs across the genome of the ISA-Nutrition participants and the 1,000 Genome Project reference data, utilizing the software PLINK 2.0 and R (SNPRelate package to control for population stratification). More details of genetic evaluation of ISA-Nutrition data are available in Pereira et al. (2024).

In our analysis, we regarded sex, age, and the first two components of global ancestry as standard covariates. Additionally, we selected variables representing lifestyle factors, biochemical markers, and health conditions acknowledged as pertinent by domain experts. Moreover, SNP data was utilized in identifying genetic anchors crucial for causal analysis among the phenotypic variables.

The variables referring to lifestyle factors, biochemical markers, and health conditions included in the analysis are described below:

#### 2.2.1 Obesity

Obesity is defined based on the body mass index (BMI), given as weight (kg)/height (m)^2^. An adult is obese if their BMI ≥ 30 kg/m^2^ (WHO Consultationm, 2000). In adolescents, obesity is identified when their BMI-for-age surpasses two standard deviations (2SD) above the mean, which, for a 19-year-old, corresponds to a BMI of 30 kg/m^2^ (Onis et al., 2007).

#### 2.2.2 Type 2 diabetes (T2D)

It is considered positive if fasting blood glucose is > 126 mg/dL or if medication for T2D (indicated by the binary variable Med_T2D) is being used, which includes hypoglycemic agents and/or insulin therapy (Cobas et al., 2022).

#### 2.2.3 Hypertension (HTN)

For adults, hypertension is diagnosed when systolic blood pressure is elevated ( ≥ 140 mmHg), diastolic blood pressure is elevated ( ≥ 90 mmHg), or if antihypertensive drugs (indicated by the binary variable Med_HTN) are being used (Barroso et al., 2021). For adolescents aged 12 and 13 years, the cutoff points for systolic or diastolic blood pressure are defined as the 95th percentile based on sex, age, and height. For individuals aged 14–19 years, the cutoffs were set at systolic blood pressure (SBP) ≥ 130 mmHg or diastolic blood pressure (DBP) ≥ 80 mmHg (Flynn et al., 2017).

#### 2.2.4 C-reactive protein (CRP)

The concentration of C-reactive proteins in the blood, obtained through a blood test, serves as a biomarker of inflammation in the body. It is measured in milligrams per deciliter (mg/dL), with the normal range typically falling below 1 mg/dL (Ridker, 2003).

#### 2.2.5 Homeostatic model assessment of insulin resistance (HOMA-IR)

HOMA-IR is calculated using the formula: fasting plasma insulin ( μ U/mL) × fasting plasma glucose (mmol/L)/22.5. The cutoff point for HOMA-1R, proposed by Geloneze et al. (2009) and validated for the Brazilian population, is set at 2.71 (Golbert et al., 2019).

#### 2.2.6 Triglycerides (TGL), LDL cholesterol (LDLc), and HDL cholesterol (HDLc)

They are all measured using a colorimetric enzymatic method with reagents from Cobas–Roche Diagnostics GmbH (Mannheim, Germany). The normal ranges are typically as follows: TGL ideally below 150 mg/dL (adults) and 130 mg/dL (adolescents), LDLc ideally below 160 mg/dL (adults) and 130 mg/dL (adolescents), and HDLc ideally above 40 mg/dL (men), 50 mg/dL (women), and 45 mg/dL (adolescents) (Précoma et al., 2019; Giuliano et al., 2005).

#### 2.2.7 Dyslipidemia (DLP)

Dyslipidemia is classified as positive if the individual is taking lipid-lowering medication (indicated by Med_DLP) or if any of the following conditions are met: elevated LDLc levels ( ≥ 160 mg/dL for adults and ≥ 130 mg/dL for adolescents), elevated TGL levels ( ≥ 150 mg/dL for adults and ≥ 130 mg/dL for adolescents), or low HDLc levels (men < 40 mg/dL, women < 50 mg/dL, and < 45 mg/dL for adolescents) (Précoma et al., 2019).

#### 2.2.8 Physical activity

A binary variable indicating whether the individual meets the total physical activity recommendations across four domains–work, domestic activities, transportation, and leisure–as outlined in the *2010 Global Recommendations on Physical Activity for Health* (World Health Organization, 2010).

Additionally, variables indicating use of Medication for T2D (Med_T2D), Medication for HTN (Med_HTN), and Medication for DLP (Med_DLP) were included in the analysis.

### 2.3 Genome-wide association study (GWAS)

Within the database of 681 unrelated individuals, we conducted a quality control process for genotypes, excluding SNPs with a minor allele frequency (MAF) of less than 5% or a Hardy-Weinberg equilibrium p-value of less than $1\times 10^{-5}$ . This process led to the removal of a total of 474,649 SNPs, resulting in a final dataset of 330,656 SNPs.

To conduct the GWAS, we included age, sex, and the square of age as covariates to separate genetic effects from the influence of these individual characteristics. Additionally, we included ancestry as a covariate using the first two estimated principal components of global ancestry to control for population structure and reduce false positive associations. We conducted a single SNP-GWAS using additive logistic regression to evaluate the association between genetic predictors and the binary phenotypic traits of interest: obesity, HTN, T2D, and DLP. For the continuous variables HDLc and CRP, we applied linear regression on their log-transformed values. In all regression models, we used hypothesis tests to determine the significance of each SNP, with a null hypothesis stating that there is no association between the SNP and the studied trait (the regression parameter associated with the SNP is equal to zero), and an alternative hypothesis stating that there is an association. To account for the increase in type I error due to multiple testing, we selected SNPs with at least genome-wide suggestive association (p-value $\leq 10^{-5}$ ).

### 2.4 AnchorFCI: causal discovery with non-ancestral knowledge

Causal discovery algorithms are increasingly being employed to elucidate, from observational data, the nature of statistical associations among variables. They can distinguish between purely spurious associations, which arise from the shared influence of other variables (referred to as confounders), and relationships that are truly causal, sometimes even elucidating their direction.

Among the existing algorithms, the Fast Causal Inference (FCI) (Spirtes et al., 2001), combined with the complete set of orientation rules by Zhang (2008b), stands out for its rigorous foundational principles and minimal reliance on assumptions compared to other methods. Since the introduction of the FCI, a range of strategies and adaptations have emerged to tackle scalability and robustness in scenarios of limited data. These include the anytime FCI by Spirtes (2001), the RFCI by Colombo et al. (2012), and their conservative counterparts (Colombo and Maathuis, 2014). Crucially, these algorithms *do not require causal sufficiency*, demonstrating their effectiveness in managing latent confounding. This capability makes FCI-like algorithms highly suitable for analyzing real-world datasets.

Relying solely on a reliable conditional independence test, the FCI and its variants learn a Partial Ancestral Graph (PAG) representing the class of all causal models that entail the set of observed conditional independencies, referred to as the Markov equivalence class (MEC). In a PAG, tails and arrowheads represent, respectively, ancestral (causal) and non-ancestral (non-causal) relationships common to all models within the most plausible MEC. A circle (“o”) denotes a non-invariant edge mark, indicating that within the MEC, there is a model where the edge mark is a tail and another model where the same edge mark is an arrowhead. Remarkably, the output is ensured to be asymptotically sound and complete, even in scenarios involving unobserved confounding and selection bias.

Scalability and integration of background knowledge are major challenges in causal discovery under latent confounding. While increasing the number of variables in the graph can boost discovery power, the reliability of the inferences often decreases due to statistical instabilities. Moreover, merely enforcing edge marks can lead to incorrect downstream orientations and violations of the equivalence class representation if the algorithm is not properly adapted. Currently, no complete strategy exists for integrating general background knowledge into the FCI framework. This integration has only been explored in specific contexts, such as studies with time series data (Gerhardus and Runge, 2020) or certain types of knowledge, such as tired (known causal order) or local knowledge (Andrews et al., 2020; Wang et al., 2022).

In this work, we introduce *anchorFCI*, a novel adaptation of the conservative RFCI designed to strategically identify reliable anchors and effectively integrate them with their known non-ancestral relationships. As detailed in Section 2.4.2, anchorFCI operates on two variable sets: the first contains the variables of interest, while the second comprises variables that are not caused by any from the first. It begins by identifying *reliable anchors*, defined as variables $V_{i}$ from the second set that are significantly associated with a variable $V_{j}$ from the first set and form unambiguous triplets during the algorithm’s conservative orientation phase. Next, it performs an adapted skeleton phase that ensures a consistent selection of d-separators while remaining order-independent, as it is based on the stable skeleton algorithm by Colombo and Maathuis (2014). Finally, it enforces arrowheads on the appropriate edges $V_{i}$ ◦−→ $V_{j}$ according to the established non-ancestral relationship between the two variable sets. Notably, strategies for managing conflicting orientations and limiting conditioning set sizes, as detailed in Section 2.4.1, are integral features of the RFCI and, thus, can be easily adjusted through parameters in the anchorFCI function. The algorithm is publicly available as an R package on GitHub at https://github.com/adele/anchorFCI and it leverages the RFCI implementation from the pcalg R package (Kalisch et al., 2024).

We apply the *anchorFCI* algorithm with strategies to enhance robustness to uncover causal relationships among the 14 phenotypic and clinical variables outlined in Section 2.2. The algorithm identifies anchors from SNPs that are significantly associated with phenotypes of interest through GWAS, and incorporates knowledge of their non-ancestral relationships. This approach not only improves robustness but also boosts discovery power by facilitating the identification of additional causal relationships. To account for confounding effects related to sex, age (both original and squared), and the first two principal components of global ancestry, these covariates will be included in all necessary tests of conditional independence.

#### 2.4.1 Strategies for addressing conflicts and inconsistencies arising from unfaithfulness

Despite the significant potential of causal discovery algorithms, they present considerable challenges in terms of scalability to larger graphs and robustness to statistical errors. Firstly, the number of potential causal structures grows super-exponentially with the number of variables, rendering the problem computationally NP-hard (Chickering et al., 2004). Achieving scalability to large graphs, particularly in scenarios involving latent confounding, necessitates reliance on model assumptions. These may include enforcing sparsity and constraining the size of conditioning sets (Claassen et al., 2013).

Moreover, the majority of the existing algorithms, including the FCI and its variants, rely on the assumption of *faithfulness*, which posits that the independencies inferred from data accurately represent the true underlying model. Notably, any falsely identified independencies can lead to conflicting orientations and inconsistencies in the implied conditional independencies (Zhang and Spirtes, 2008; Zhalama et al., 2017). This poses a significant challenge for real-world applications, particularly when dealing with limited sample sizes, as statistical tests may lack sufficient power to accurately identify potentially weak or noisy associations. This issue becomes more pronounced when dealing with larger graphs (e.g., 10 or more variables), exemplifying the curse of dimensionality, as it leads to an increased number of conditional independence tests and a significant decrease in statistical power as the conditioning set size grows.

To minimize issues arising from unfaithfulness, we employ the following two strategies:

• Conservative edge orientations with majority rule: as proposed by Colombo and Maathuis (2014), edge orientations are strictly conducted conservatively, meaning that they exclusively rely on triplets that have been previously determined as unambiguous. To assess the ambiguity of an unshielded triplet (i.e., three variables where the first and second, and the second and third, are adjacent, but not the first and third), additional conditional independence tests are performed. These tests aim to detect errors in determining whether the second variable belongs or not to the set that renders the first and third variables independent of each other, potentially due to instances of unfaithfulness. With the majority rule approach, the decision is based on the majority of the involved conditional independence tests. In the event of a tie, the triplet is deemed ambiguous, and no orientation is conducted.
• Constrained conditioning set size: as pointed out by Spirtes (2001) and Colombo et al. (2012), the accuracy of the FCI significantly decreases when relying on independencies conditional on a large set of variables, as, in such cases, statistical errors due to the low power of the tests become inevitable, especially with small sample sizes. They also demonstrate that restricting the size of conditioning sets in the independence tests does not compromise the correctness of the output PAG; it may only lead to a less informative PAG. In light of this result, the RFCI not only accepts a constraint on the size of the conditioning sets, but also employs a procedure that requires fewer conditional independence tests than the FCI. The authors demonstrated the RFCI’s consistency in sparse scenarios, showing that by avoiding statistical tests with the least power (i.e., those with relatively large conditioning sets), the results become notably more reliable, particularly for small sample sizes.

#### 2.4.2 Identification of reliable anchors and integration of non-ancestral knowledge

Applying the RFCI combined with strategies aimed at enhancing robustness (e.g., conservative edge orientations) can contribute to more reliable results. However, these strategies often result in PAGs with numerous underdetermined edge marks (i.e., circles).

To enhance robustness and discovery power, we propose extending the RFCI algorithm to utilize two partially ordered variable sets: the first contains the variables of interest, while the second comprises variables known not to be caused by any variable in the first set. This structure is ideal for datasets with non-genetic variables, such as phenotypic, clinical, or sociodemographic data, in the first set, and genetic variables in the second. This novel algorithm, referred to as *anchorFCI*, introduces three key adaptations:

1. Identification of robust anchors: These are variables from the second set (e.g., SNPs) that are identified as significantly associated with variables from the first set (e.g., phenotypes) and, upon integration into the graph, form triplets that are unambiguous according to the majority rule.
2. Skeleton inference with an adapted search for minimal d-separating sets: In the initial phase of the algorithm, known as the adjacency phase, the model’s skeleton is inferred through a series of conditional independence tests. Whenever a pair of variables is found to be conditionally independent given a set of other variables, such separating set is considered minimal and the edge connecting them is removed. In this phase, it is essential to prevent the algorithm from performing independence tests between two variables from the second set (e.g., two SNPs) conditional on any variable from the first set (e.g., a phenotype). Notably, this restriction does not compromise the correctness or completeness of the algorithm. This can be demonstrated through a direct application of (Tian et al., 1998, Theorem 2), which states that if a set Z d-separates two variables X and Y , we can get a smaller d-separator $Z^{\prime }$ by removing from Z all nodes that are not ancestors of X or Y . This implies that, since the first set consists entirely of non-ancestors of the second set, any faithful minimal separating set for a pair of variables from the second set will necessarily exclude variables from the first set.
3. Enforcing known arrowheads: During the orientation phase of the algorithm, edge-mark inference rules (R0 to R10, as detailed in Zhang (2008b)) are sequentially applied until they can no longer be utilized. Throughout this phase, we ensure that every edge connecting a variable $V_{i}$ from the first set (e.g., a SNP) and a variable $V_{j}$ from the second set (e.g., a phenotype) is oriented with an arrowhead pointing towards $V_{j}$ . In other words, these edges will take the form $V_{i}*\to V_{j}$ , where ∗ denotes any edge mark that the algorithm may learn. Incorporating this type of background knowledge is straightforward and can be achieved simply by applying the rules once the known edge marks are enforced and then checking the validity of the PAG as an ancestral graph (i.e., no cycles or almost cycles). Notably, the integration of non-ancestral knowledge in the context of time-series data (Gerhardus and Runge, 2020) showcases the potential to enhance both robustness and learning capabilities in causal discovery.

Notably, when applied to a dataset comprising SNPs and phenotype variables, the resultant PAG from the anchorFCI algorithm will exclusively include edges between an SNP and a phenotype falling into one of the following types: SNP ↔ Phenotype (indicating purely spurious association), SNP → Phenotype (indicating a causal SNP), or SNP ◦→ Phenotype (indicating there is not sufficient evidence to determine whether the SNP is a cause or only spuriously associated with the phenotype).

By integrating inherently unambiguous orientations, anchorFCI not only prevents discrepancies with established non-ancestral knowledge, potentially induced by violations of faithfulness, but also boosts discovery power by facilitating the conservative identification of additional causal relationships.

**FIGURE 1:**
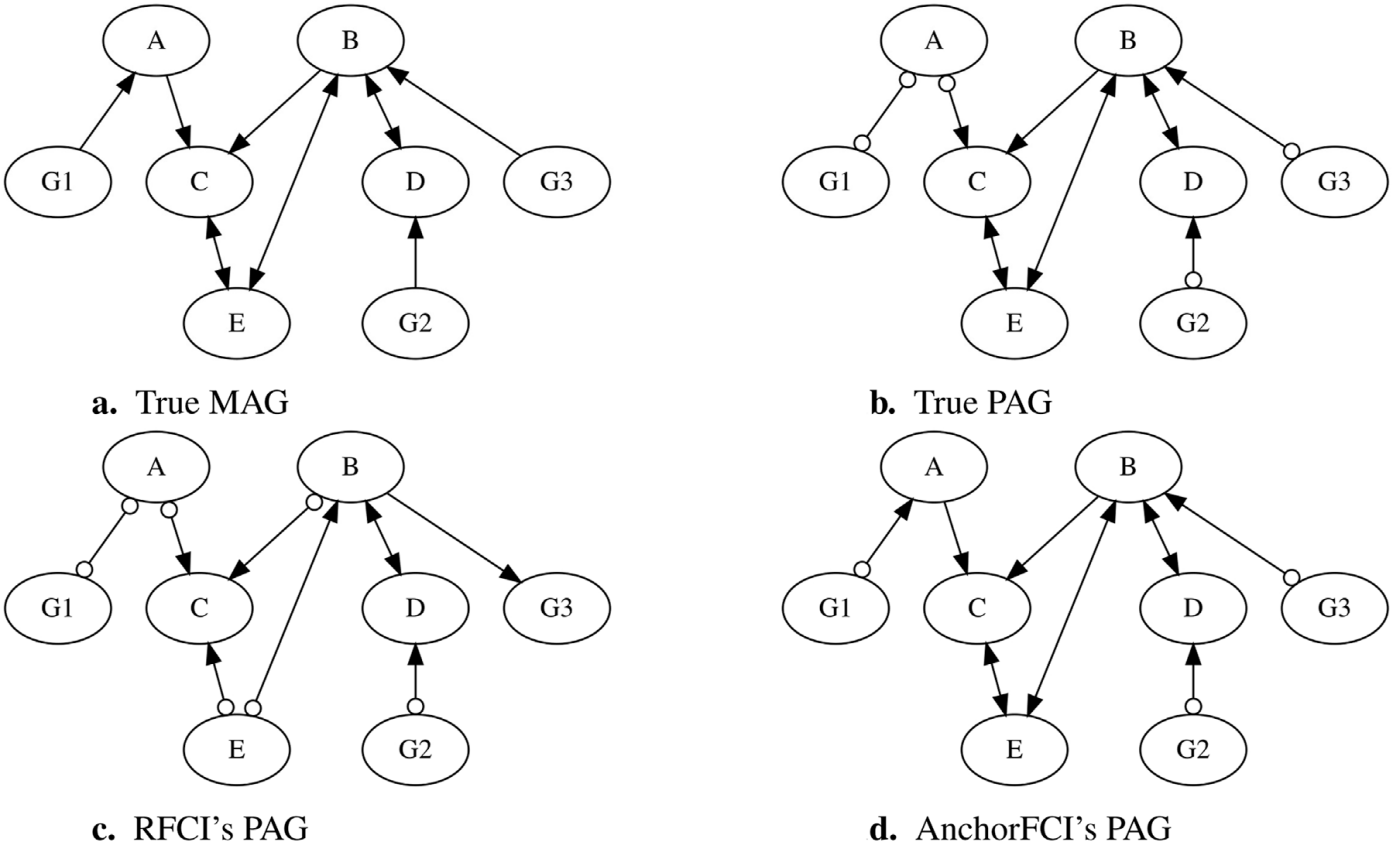
Comparison of PAGs inferred by RFCI and anchorFCI using a simulated dataset. The dataset was generated based on the MAG in **(A)**, structured as $G_{1},G_{2},G_{3}≺A,B,C,D,E$ . The true PAG is presented in **(B)**, while the PAGs inferred by RFCI and anchorFCI are shown in **(C, D)**, respectively.

To further illustrate how anchorFCI can achieve greater robustness and accuracy compared to RFCI, particularly in limited data settings where the faithfulness assumption is likely to be violated, we selected a dataset from our simulation study, as detailed in Section 2.4.4. The dataset consists of 1,000 samples, generated based on the Maximal Ancestral Graph (MAG) shown in Figure 1A. The variables $\left\{G_{1},G_{2},G_{3}\right\}≺\left\{A,B,C,D,E\right\}$ , with ≺ denoting precedence in the partial ordering. The corresponding true PAG, obtained without using the partial order information, is shown in Figure 1B.

When applying the conservative RFCI algorithm with the majority rule, we obtain the PAG in Figure 1C. The collider triplets ⟨A,C,B⟩ , ⟨E,B,D⟩ , ⟨A,C,E⟩ , and $\left\langle G_{2},D,B\right\rangle$ are identified as unambiguous and correctly oriented. The triplets $\left\langle G_{1},A,C\right\rangle$ and $\left\langle G_{3},B,C\right\rangle$ are identified as unambiguous non-collider triplets, however, without the partial order information, their orientations cannot be determined. The triplet ⟨C,B,D⟩ is marked as ambiguous. After applying the remaining orientation rules, RFCI incorrectly orients $B\to G_{3}$ , violating the known, but unused, information that $G_{3}≺B$ . The resulting PAG has a Structural Hamming Distance (SHD) of five from the true PAG, reflecting the number of incorrect orientations.

In contrast, when applying anchorFCI, we obtain the PAG in Figure 1D, which not only accurately captures all the oriented edges from the true PAG but also correctly identifies additional orientations from the original MAG. First, variables $G_{1}$ , $G_{2}$ , and $G_{3}$ are all identified as reliable anchors, as they form unambiguous triplets $\left\langle G_{1},A,C\right\rangle$ , $\left\langle G_{2},D,B\right\rangle$ , and $\left\langle G_{3},B,C\right\rangle$ . Then, after applying the adapted skeleton algorithm, anchorFCI integrates the known non-ancestral relationships by orienting $G_{1}◦\to A$ , $G_{2}◦\to D$ , and $G_{3}◦\to B$ . This not only avoids the mistakes made by RFCI but also enables the accurate orientation of the edge B→C and of the collider ⟨C,E,B⟩ . Notably, anchorFCI also infers the tail on A→C , indicating that A is a definite cause of C , thereby learning a representation that goes beyond the MEC.

#### 2.5 Conditional independence tests for mixed data

As stated before, the FCI and its variants identify ancestral and non-ancestral relationships by employing a combination of conditional independence tests and orientation rules.

To test conditional independence between binary (obesity, T2D, HTN, DLP, physical activity, and medication variables), continuous (CRP, HOMA-IR, TGL, LDLc, and HDLc), and multinomial (SNP) variables, we use the symmetric conditional independence test for mixed data proposed by Tsagris et al. (2018), available in the MXM R package (Lagani et al., 2017). This test utilizes linear or generalized linear regression, depending on the nature of the involved variables. Logistic regression is employed for binary outcomes, Gaussian linear regression for continuous outcomes, and multinomial log-linear regression for multinomial outcomes. To prevent departures from normality in tests involving continuous variables, we transformed them using the rank-based inverse normal transformation available in the RNOmni R package (McCaw, 2023). Missing values were imputed using *MissForest*, a non-parametric method for imputing mixed-type data sets by employing random forests (Stekhoven and Bühlmann, 2012). We utilized the implementation of the MissForest algorithm available in the MissRanger R package (Mayer and Mayer, 2019), setting the number of forests to 500.

Formally, Tsagris et al. (2018) test evaluates the conditional independence of two variables, $V_{i}$ and $V_{j}$ , given a set of variables $S_{ij}$ , by testing two null hypotheses: $H0_{1}:P\left(V_{i}|S_{ij}\right)=P\left(V_{i}|V_{j},S_{ij}\right)$ and $H0_{2}:P\left(V_{j}|S_{ij}\right)=P\left(V_{j}|V_{i},S_{ij}\right)$ . The null hypothesis $H0_{1}$ is tested using a nested likelihood-ratio test comparing a reduced model (where $V_{i}$ is regressed on $S_{ij}$ ) against a full model (where $V_{i}$ is regressed on both $S_{ij}$ and $V_{j}$ ). Similarly, $H0_{2}$ is tested by reversing the roles of $V_{i}$ and $V_{j}$ . In general, the p-values $p_{1}$ and $p_{2}$ from the tests for $H0_{1}$ and $H0_{2}$ , respectively, tend to be identical only asymptotically. To correct for any potential asymmetry in limited data scenarios, we adopt Tsagris et al. (2018) strategy of merging dependent p-values. Such method calculates the combined p-value as $\text{min}\left(2\times \text{min}\left(p_{1},p_{2}\right),\text{max}\left(p_{1},p_{2}\right)\right)$ and has demonstrated superior learning accuracy when compared to alternative methods.

All tests include sex, age (original and squared), and the first two principal components of global ancestry in the conditioning set, ensuring comprehensive adjustment for these variables. Importantly, these variables stand out as special covariates because they are not caused by any other variables, and thus, conditioning on them can never introduce biases such as collider bias (Holmberg and Andersen, 2022).

Finally, all tests were conducted with a significance level set at 5%, without applying any correction for multiple comparisons. In contrast to association studies, which aim to validate statistical *dependencies*, causal discovery relies on establishing reliable statistical *independencies*. It is crucial to note that the non-rejection of the null hypothesis (indicating conditional independence) does not imply the acceptance of the alternative hypothesis (indicating conditional dependence). Any correction aimed at reducing the number of falsely identified associations may significantly increase the number of falsely identified independencies, thereby triggering a cascade of erroneous edge orientations. Conversely, as noted by Spirtes (2001); Colombo et al. (2012), false associations often prevent certain edge orientations, leading to a final PAG that, while less informative, still tends to uphold accuracy.

#### 2.6 Comparative study of RFCI and AnchorFCI

To quantitatively assess the enhanced robustness and discovery power of anchorFCI compared to the conservative RFCI, we designed a simulation study. To capture diverse scenarios, we simulated 50 unique random MAGs with eight nodes, structured as $\left\{G_{1},G_{2},G_{3}\right\}≺\left\{A,B,C,D,E\right\}$ . Additionally, to account for varying degrees of unfaithfulness, we generated 30 datasets for each MAG, with sample sizes N=500,1000,5000,10000 . The variables $G_{1},G_{2},G_{3}$ are modeled as discrete variables, each with three levels, following a multinomial distribution, while A,B,C,D,E are continuous variables, following a Gaussian distribution. The datasets were generated using the simMixedDAG R package (Lin, 2019), where each variable is modeled as a linear combination of its parents (including potential latent variables), with randomly assigned coefficients. The choice of distributions does not impact the comparison between the algorithms but was selected to reflect typical applications involving genotype and phenotype variables.

Since anchorFCI aims to enhance causal discovery among the variables of interest {A,B,C,D,E} by selecting reliable anchors from $\left\{G_{1},G_{2},G_{3}\right\}$ , we evaluate the algorithms using a score based on the Structural Hamming Distance (SHD) computed considering exclusively edges among the variables of interest. Given that anchorFCI can identify orientations beyond those in the true MEC’s PAG (i.e., the one obtained without using any partial order information), this score allows us to assess both the accuracy and informativeness of the inferred PAG. It is calculated as the difference between the SHD of the inferred PAG relative to the true MAG, and the SHD of the true MEC’s PAG relative to the true MAG. Smaller scores indicate higher accuracy, with zero indicating the inferred PAG is as informative as the true MEC’s PAG (i.e., the one obtained without using any partial order information), and negative values indicating the inferred PAG is more informative than the true MEC’s PAG.

### 2.7 Causal effect identification and estimation

As previously discussed, a PAG represents a class of the most probable models based on the available observational data. Remarkably, all models in this class fit the data equally well, as they entail the same set of observed conditional independencies, rendering them statistically indistinguishable. Whenever ancestral and non-ancestral relationships are shared across all models within this equivalence class, they are represented as non-circle edge marks in the PAG. While this may suffice for a qualitative description of the relationships among the variables, a more comprehensive approach is required to quantify a causal effect.

For each directed edge inferred in the PAG, it is necessary to assess the identifiability of the corresponding causal effect. The identification of a causal effect is contingent upon its uniqueness–it is identifiable if and only if it is uniquely computable among all models within the equivalence class represented by the PAG, and utilizing the same expression solely based on observational (conditional) probabilities.

A necessary condition for identifying the causal effect of a variable X on a variable Y is that the edge X→Y is visible, indicating that X and Y do not share any latent causes This condition can be easily verified through a graphical criterion presented by Zhang (2008a).

The generalized backdoor criterion (Maathuis and Colombo, 2015) is regarded as one of the most straightforward effect identification graphical criteria. It states that the causal effect of a variable X on a variable Y can be identified from a PAG P if there exists a set Z , comprising solely non-descendants of X , that blocks (in the sense of d-separation–see Pearl (2000b)) all definite-status backdoor (confounding) paths between X and Y in P . In this case, the post-interventional probability distribution of a variable Y after an intervention that sets X=x , denoted do(X=x) , is expressed by:

$$P\left(Y=y|do\left(X=x\right)\right)=\int_{z}P\left(Y=y|X=x,Z=z\right)P\left(Z=z\right)dz.$$

Such an integral can be estimated from N observational samples ${\left\{y_{i},x_{i},z_{i}\right\}}_{i=1}^{N}$ as following:

$$\hat{P}\left(Y=y|do\left(X=x\right)\right)=\overset{N}{\underset{i=1}{\sum }}\hat{f}\left(x,z_{i}\right),$$

where $\hat{f}\left(x,z\right)=\hat{P}\left(Y=y|X=x,Z=z\right)$ can be readily obtained through (generalized) regression analysis of Y on X and Z . Note that if Z=∅ is admissible for backdoor adjustment, the interventional distribution simplifies to P(Y=y|do(X=x))=P(Y=y|X=x) , which can also be readily obtained using (generalized) regression analysis of Y on X . If continuous response variables are (natural) log-transformed before regression analyses to normalize residual distributions, then estimates are subsequently back-transformed to the original scale using the improved Cox’s method proposed by Olsson (2005). Effect sample sizes for each prediction are determined using the methodology by Thomassen et al. (2024).

In cases where the generalized backdoor criterion does not apply, alternative tools for causal effect identification and estimation from PAGs are available. These include the generalized adjustment criterion, introduced by Perkovi et al. (2018), and the complete causal calculus and (conditional) complete effect identification algorithm developed by Jaber et al. (2022).

## 3 Results

### 3.1 Descriptive analysis

In the database of 681 individuals, the proportions of gender are similar, with 53.59% male participants, and the mean age is 44.70 ± 23.37 years. The age groups also have similar proportions: 29.51% of adolescents (12–19 years old), 32.74% adults (20–59 years old), and 37.73% older adults ( ≥ 60 years old).

The proportions of binary health conditions vary: 23.2% of individuals are obese, 14.7% have T2D (with 70% of them taking medication for T2D), 45.8% have HTN (with 53.4% of them taking medication for HTN), and 66.7% have DLP (with 12.8% of them taking medication for DLP). Moreover, 68.8% of individuals fulfill the 2010 WHO recommendations for total physical activity (Bull et al., 2020). Summary statistics for the continuous variables in the study are provided in Table 1.

**TABLE 1:** Summary statistics for the continuous variables in the study, including Minimum (Min.), Median, Mean, 1st and 3rd Quantiles (Qu.), Maximum, and total number of missing values (NA’s).

	Min.	1st Qu.	Median	Mean	3rd Qu.	Max.	NA’s
CRP (mg/dL)	0.02	0.10	0.30	0.54	0.78	2.24	21
HOMA-IR	0.35	1.76	2.69	3.58	4.08	33.89	8
HDLc (mg/dL)	10.00	35.00	43.00	44.14	52.00	119.00	14
LDLc (mg/dL)	6.00	77.50	101.00	103.52	126.00	259.00	14
TGL (mg/dL)	10.00	73.00	100.00	120.22	139.50	1402.00	13

### 3.2 Genome-wide association study (GWAS)

We conducted a single SNP-GWAS to detect the association between each individual SNP and six phenotypic traits: obesity, HTN, T2D, DLP, CRP levels, and HDLc. Utilizing a p-value threshold indicative of genome-wide suggestion, set at $10^{-5}$ , we identified a total of ten SNPs.

**FIGURE 2:**
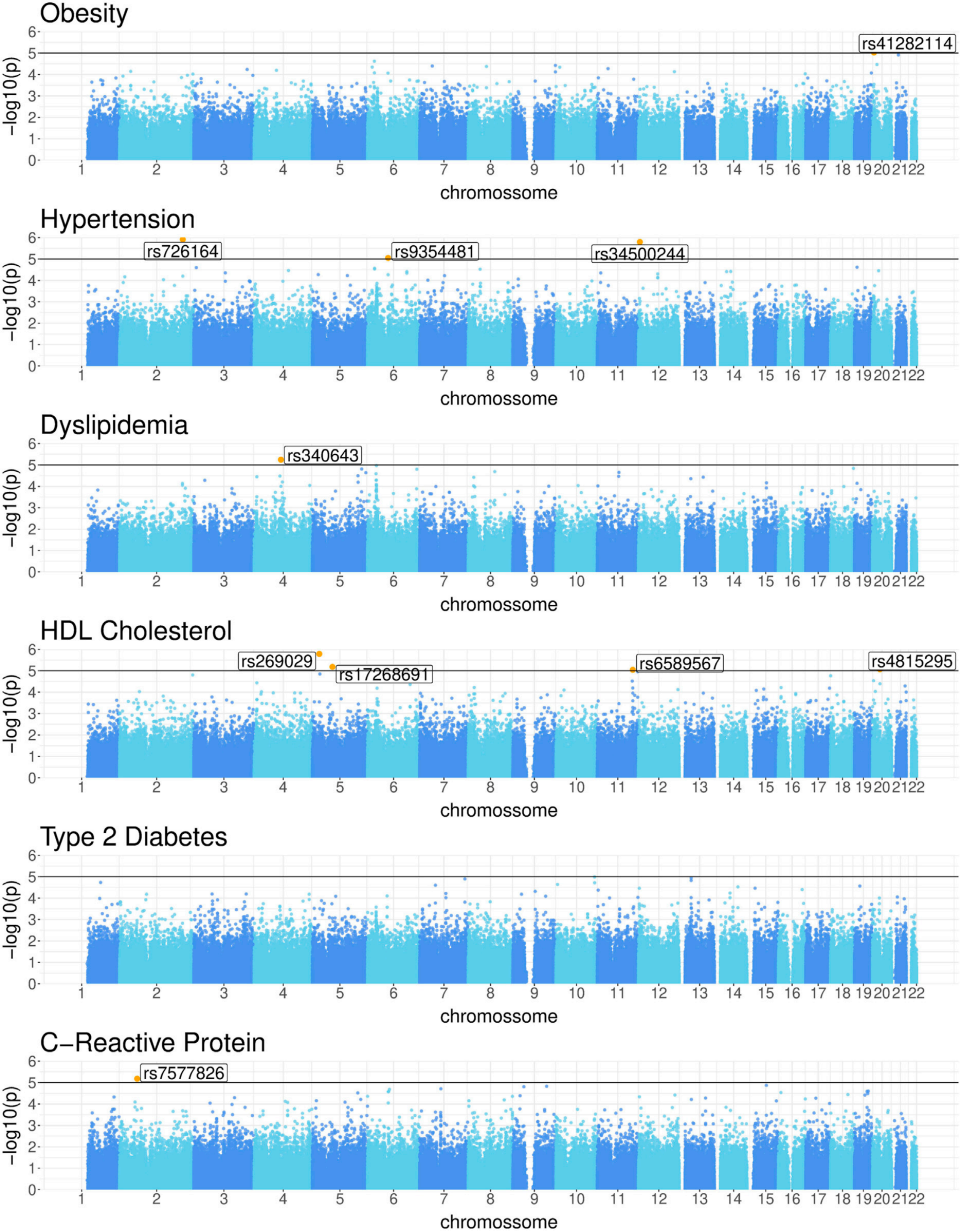
Manhattan plots for Obesity, Hypertension, Dyslipidemia, HDL cholesterol, Type 2 Diabetes, and C-Reactive Protein levels.

Specifically, the Manhattan plots in Figure 2 reveal the following genetic associations: 1 SNP for obesity (rs41282114), 3 for HTN (rs726164, rs34500244, rs9354481), 1 for DLP (rs340643), 4 for HDLc (rs269029, rs17268691, rs6589567, rs4815295), and 1 for CRP levels (rs7577826). No genetic associations with T2D were found at this threshold. Table 2 presents a detailed summary of the SNPs identified for each of the phenotypic traits studied. Notably, no SNP was found to be common across all phenotypic traits. Observe that the SNPs associated with DLP, HDL, and CRP exhibit negative effects, indicating an association with a reduction in the respective studied traits. These identified SNPs are potential genetic markers for the respective conditions and may contribute to our understanding of the underlying genetic architecture. Additionally, they will undergo further analysis in the causal discovery phase to determine their potential as reliable anchors, thereby aiding in learning of causal relationships.

**TABLE 2:** List of SNPs associated with Obesity, HTN, and DLP, HDL and CRP at a significance level of $10^{-5}$ , with their corresponding minor allele, MAF, chromosome (Chr.), linear regression coefficient (β) , and p-value.

Trait	SNP	Minor allele	MAF	Chr.	β	p-value
Obesity	rs41282114	T	0.07	20	0.98	$9.3\times 10^{-6}$
HTN	rs726164	A	0.45	2	0.65	$1.2\times 10^{-6}$
	rs34500244	.	0.07	12	1.40	$1.6\times 10^{-6}$
	rs9354481	G	0.12	6	−0.62	$9.0\times 10^{-6}$
DLP	rs340643	A	0.36	4	−0.57	$5.7\times 10^{-6}$
HDLc	rs269029	G	0.29	5	−0.09	$1.6\times 10^{-6}$
	rs17268691	T	0.10	5	−0.13	$6.6\times 10^{-6}$
	rs6589567	A	0.12	11	−0.11	$9.0\times 10^{-6}$
	rs4815295	T	0.41	20	−0.08	$8.5\times 10^{-6}$
CRP	rs7577826	C	0.34	2	−0.30	$6.6\times 10^{-6}$

### 3.3 Causal discovery using the proposed AnchorFCI algorithm

#### 3.3.1 Comparative study of RFCI and AnchorFCI

The results of the simulation study comparing the performance of RFCI and anchorFCI in learning the structure among the variables of interest are summarized in Table 3. The analysis reveals that anchorFCI consistently exhibits superior robustness and accuracy compared to RFCI. This is supported by statistically significantly lower scores for anchorFCI, with all p-values from a one-sided Wilcoxon test indicating very strong significance. Additionally, anchorFCI exhibits enhanced discovery power. Notably, it often achieves negative scores, indicating its ability to learn representations beyond the MEC even in scenarios with limited data. While both methods improve in performance with increasing sample sizes, anchorFCI consistently outperforms RFCI across all evaluated conditions.

**TABLE 3:** Comparison of RFCI and anchorFCI performance in learning the structure among the variables of interest. Scores represent the difference between the SHD of the inferred PAG relative to the true MAG and the SHD of the true PAG relative to the true MAG.

N	RFCI		AnchorFCI			Diff	P-value
	[Min, max]	Mean ± SD	[Min, max]	Mean ± SD	#Anchors		
500	[2.00, 7.40]	5.33 ± 1.27	[−0.53, 6.30]	3.72 ± 1.39	1.84	1.62	$8.76\times 10^{-193}$
1,000	[1.47, 5.97]	4.27 ± 1.12	[−0.80, 4.43]	2.72 ± 1.28	2.19	1.55	$4.94\times 10^{-204}$
5,000	[0.80, 3.83]	2.65 ± 0.805	[−1.60, 2.83]	1.19 ± 1.10	2.64	1.47	$9.63\times 10^{-227}$
10,000	[0.60, 3.57]	2.21 ± 0.716	[−2.20, 2.40]	0.70 ± 0.97	2.73	1.50	$8.07\times 10^{-236}$

Values are averages across 50 randomly generated MAGs, each evaluated on 30 datasets with varying sample sizes N . #Anchors indicates the average number of selected reliable anchors by anchorFCI. Smaller scores indicate higher accuracy, with zero indicating performance equivalent to the true PAG and negative scores suggesting greater informativeness beyond the MEC. P-values are from a one-sided Wilcoxon test with alternative hypothesis that the average difference between RFCI and anchorFCI scores (Diff) is greater than 0.

For completeness, we provide a comparison of performance in learning the graphical structure among the variables of interest and the anchors in the Supplementary Material. As expected, anchorFCI significantly outperforms RFCI, thanks to its effective integration of partial order knowledge. Notably, while RFCI exhibits considerable instability in low-data regimes, anchorFCI consistently demonstrates superior robustness and accuracy.

#### 3.3.2 Applying AnchorFCI to the 2015 ISA-nutrition dataset

**FIGURE 3:**
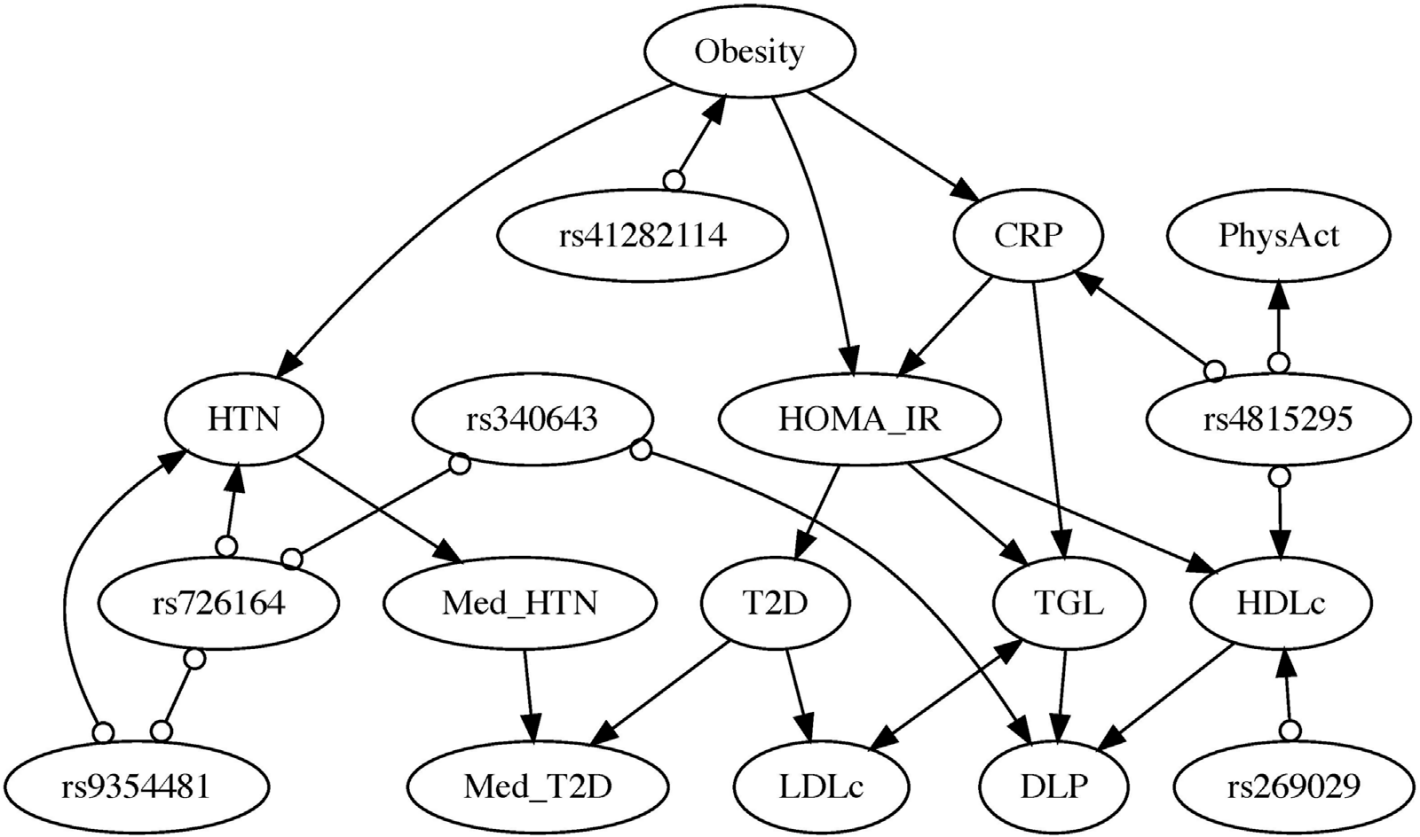
Partial Ancestral Graph inferred by the anchorFCI algorithm, using conservative orientations guided by the majority rule, and integrating the partial order SNPs ≺ phenotype variables. Directed edges imply ancestral (causal) relationships. Bidirected edges imply purely spurious associations. Circles indicate uncertain edge-marks, interchangeable with tail or arrowhead in equally probable models. Nodes representing various phenotypic traits include: Obesity, Type 2 Diabetes (T2D), Hypertension (HTN), Dyslipidemia (DLP), C-reactive protein (CRP), Homeostatic Model Assessment of Insulin Resistance (HOMA-IR), Triglycerides (TGL), Low-Density Lipoprotein Cholesterol (LDLc), High-Density Lipoprotein Cholesterol (HDLc), Medication for T2D (Med_T2D), Medication for HTN (Med_HTN), and Physical Activity (PhysAct).

Figure 3 shows the output PAG from our proposed anchorFCI algorithm. Conditional independence for mixed variables were conducted following the approach in Section 2.4.3, at a significance level of 5%. Furthermore, as outlined in Section 2.4.1, we enforced a limitation on the size of the conditioning sets, allowing for a maximum of 2 (two) variables. This choice is based on our exhaustive experimentation with the algorithm, which revealed that independencies conditional to larger sets led to the separation of certain pairs of dependent variables within the graph, thereby compromising the model’s accuracy. Edge mark inference was conducted conservatively, meaning that edge orientations were established solely based on triplets identified as unambiguous according to the majority rule.

Among all SNPs identified as significantly associated with phenotypes (with p-value less than $10^{-5}$ ), the anchorFCI algorithm exclusively selected those forming unambiguous triples in the inferred PAG, as determined by the majority rule. These include: rs41282114 (associated with Obesity), rs726164 and rs9354481 (associated with HTN), rs340643 (associated with DLP), and rs269029 and rs4815295 (associated with HDLc). Prior knowledge asserting those SNP variables cannot be caused by any of the other variables was incorporated in the learning process through the steps detailed in Section 2.4.2. Note that we excluded Med_DLP from the analysis due to numerous conditional independence tests raising errors, primarily because of the limited number of individuals who take medication for DLP in our sample.

In the resulting PAG, the relationships among all phenotypes and clinical variables have been fully characterized. Only a few edge marks in edges with SNPs remain undetermined (i.e., circles).

Directed edges represent definite ancestral (causal) relationships. Obesity emerges as a significant upstream causal factor for multiple conditions. It directly influences HTN, CRP levels–suggesting an impact on the body’s inflammation levels –, and insulin resistance, as measured by HOMA-IR. Insulin resistance, inferred to be directly influenced by CRP, is considered a causal factor for T2D, TGL, and HDLc. TGL and HDLc are identified as causes of DLP. Furthermore, T2D appears to causally contribute to LDLc. The probabilities of undergoing medication for HTN (Med_HTN) and for T2D (Med_T2D) are, as expected, influenced by their respective conditions. Med_HTN appears to influence the likelihood of Med_T2D. Bidirected edges represent definite spurious associations, indicating no causal relationship in any direction. Notably, the model suggests that the relationship between LDLc and TGL exhibits this characteristic, thus solely attributable to the influence of latent confounders.

To assess the stability of the inferred relationships, we present the results from 50 bootstrap samples in the Supplementary Material. The following relationships were inferred in the respective percentages of bootstrap samples: Obesity → HOMA-IR (88%), TGL → DLP (84%), HTN → Medication for HTN (80%), TGL ↔ LDLc (64%), HDLc → DLP (58%), Medication for HTN → Medication for T2D (58%), HOMA-IR → T2D (54%). For certain relationships, however, the highest percentages correspond to non-adjacency, suggesting weak connections between CRP and TGL (52%), HOMA-IR and HDLc (42%), and Obesity and HTN (38%). For the remaining relationships, the edge types inferred with highest percentage correspond to those inferred from the original dataset, although such percentage did not exceed 50%.

It is essential to emphasize that the stability of any causal discovery algorithms is highly dependent on the number of variables in the graph, with performance significantly declining when the number of variables exceeds a handful. To illustrate this point, we present bootstrap results in the Supplementary Material based on only nine variables: Obesity, HOMA-IR, T2D, LDLc, HDLc, TGL, DLP, rs41282114, and rs4815295. In this scenario, the results are significantly more robust, as exemplified by the following relationships and respective percentages of bootstrap samples: Obesity → HOMA-IR (100%), TGL → DLP (90%), HOMA-IR → T2D (82%), and T2D → LDLc (54%).

Notably, if we had employed the original conservative RFCI with the SNP variables but without the adaptations proposed in the anchorFCI algorithm, not only would numerous orientations remain undetermined, but we would also see some phenotypes identified as causes of genotypes (e.g., DLP is learned as a cause of the SNP rs340643), contradicting the current understanding that genetic variables cannot be caused by phenotypes. For further details, please refer to the Supplementary Material.

### 3.4 Causal effect identification and estimation

All directed edges in Figure 3 of the PAG are free from latent confounding influence (as per the visibility graphical criterion by Zhang (2008a)), and their corresponding total causal effects are all identified using the generalized backdoor criterion (GBDC), by Maathuis and Colombo (2015). The estimation of interventional expectations and probabilities was conducted as detailed in Section 2.5. For coefficient estimates, associated statistics, and p-values of all regression models involved, please refer to the Supplementary Material.

#### 3.4.1 Obesity → HTN

**FIGURE 4:**
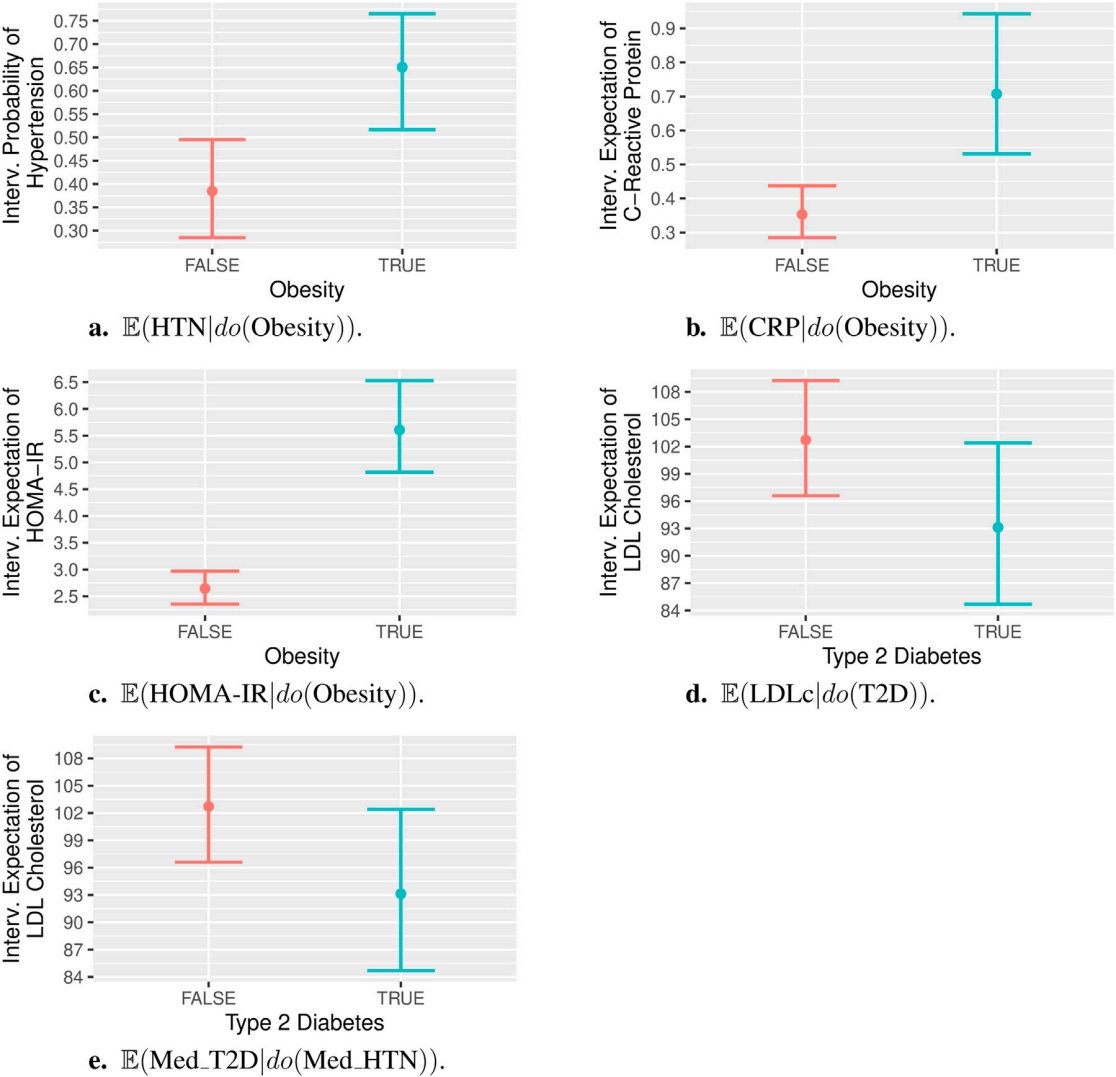
Post-interventional probabilities/expectations, with interventions on binary variables. Specifically, panels **(A)** to **(E)** show: E(HTN|do(Obesity)) , E(CRP|do(Obesity)) , E(HOMA-IR|do(Obesity)) , E(LDLc|do(T2D)) , and E (Med_T2D|do(Med_HTN)) . Dots represent point estimates, while the error bars indicate the 95% confidence intervals.

Figure 4A shows the point estimate and 95% confidence interval (CI) for the probability of HTN given an intervention on Obesity (P(HTN|do(Obesity))) . The effect appears as unconfounded in the PAG, so the adjustment only considered the standard set of covariates, namely sex, age (original and squared), and the first two principal components of global ancestry. The non-overlapping intervals suggest a significant difference between these probabilities: for non-obese individuals, it is 0.38 with a 95% CI of [0.29, 0.5], while for obese individuals, it is 0.65 with a 95% CI of [0.52, 0.76].

#### 3.4.2 Obesity → CRP

Figure 4B shows the point estimate and 95% CI for the expectation of CRP levels given an intervention on Obesity (E(CRP|do(Obesity))) . Since the effect appears unconfounded in the PAG, we adjusted solely for the standard set of covariates. The difference between these expectations is significant: for non-obese individuals, it is 0.35 with a 95% CI of [0.28, 0.44], while for obese individuals, it is 0.71 with a 95% CI of [0.53, 0.94].

#### 3.4.3 Obesity → HOMA-IR

Figure 4C shows the point estimate and 95% CI for the expectation of HOMA-IR levels given an intervention on Obesity (E(HOMA-IR|do(Obesity))) . Once more, the estimated expectations were adjusted solely by the standard set of covariates, and the difference between the expectations is statistically significant: for non-obese individuals, it is 2.6 with a 95% CI of [2.4, 3], while for obese individuals, it is 5.6 with a 95% CI of [4.8, 6.5].

#### 3.4.4 T2D → LDLc

Figure 4D shows the point estimate and 95% CI for the expectation of LDLc given an intervention on T2D (E(LDLc|do(T2D))) . Given that the effect appears unconfounded in the PAG, it was adjusted solely by the standard set of covariates. For individuals without T2D, it is 100 with a 95% CI of [97, 110], while for individuals with T2D, it is 93 with a 95% CI of [85, 100]. The overlapping of the confidence intervals prevents us from concluding that the difference is significant.

#### 3.4.5 Medication for HTN → medication for T2D

Figure 4E shows the point estimate and 95% CI for the probability of taking medication for T2D given an intervention on medication for HTN (P(Med_T2D|do(Med_HTN))) . In accordance with the GBDC, the unbiased effect is achieved through adjustment for Obesity and standard covariates. If an individual is on medication for HTN, the probability of taking medication for T2D is 0.02, with a 95% CI of [0.0061, 0.064]. Conversely, if an individual is not on medication for HTN, the probability is 0.15, with a 95% CI of [0.05, 0.36]. Again, the overlapping of the confidence intervals prevents us from concluding that the difference is significant.

#### 3.4.6 CRP → HOMA-IR

**FIGURE 5:**
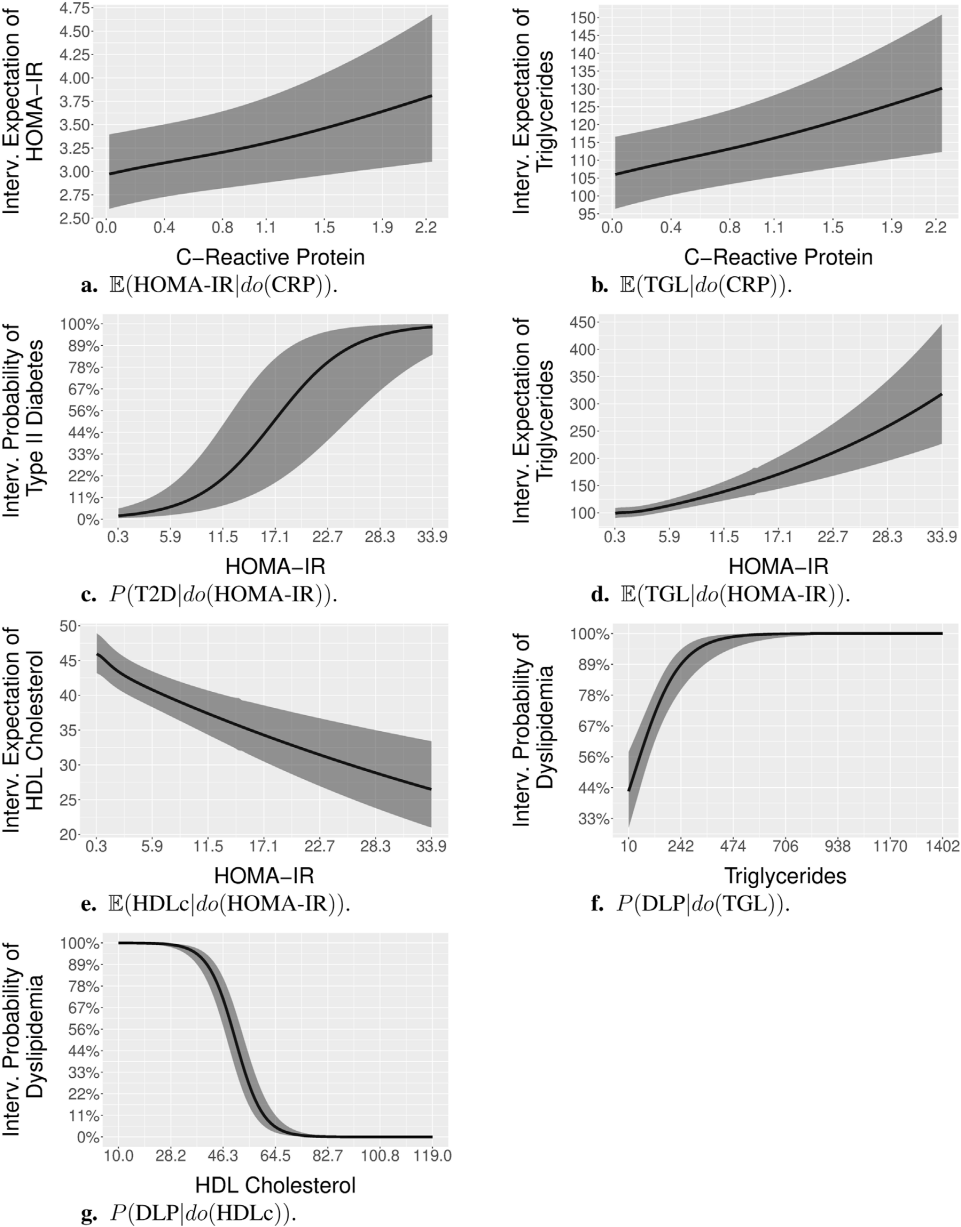
Post-interventional probabilities and expectations, with interventions on continuous variables. Specifically, panels (**A)**. to (**G)**. show: E(HOMA-IR|do(CRP)) , E(TGL|do(CRP)) , P(T2D|do(HOMA-IR)) , E(TGL|do(HOMA-IR)) , E(HDLc|do(HOMA-IR)) , P(DLP|do(TLG)) , and P(DLP|do(HDLc)) . Full lines represent average estimates, while the shaded areas indicate the 95% confidence regions.

Figure 5A shows the average estimate and 95% confidence region for the expectation of HOMA-IR levels given different levels of CRP set by intervention (E(HOMA-IR|do(CRP))) . As per GBDC, estimates were obtained by adjusting for Obesity along with the standard set of covariates. While, on average, HOMA-IR levels tend to increase with CRP levels, the confidence region, which includes the constant line, suggests that this trend may not be statistically significant. For individuals with CRP = 0.3, the post-interventional expectation of HOMA-IR is 3.1, with a 95% CI of [2.7, 3.5]. In contrast, for individuals with CRP = 2, the expectation is 3.7, with a 95% CI of [3.1, 4.5].

#### 3.4.7 CRP → TGL

Figure 5B shows the average estimate and 95% confidence region for the expectation of TGL given different levels of CRP set by intervention (E(TGL|do(CRP))) . As per GBDC, estimates were obtained by adjusting for Obesity along with the standard set of covariates. On average, TGL levels tend to rise with increasing CRP levels. However, the confidence region, which includes the constant line, suggests that this trend may not be statistically significant. Specifically, for individuals with CRP = 0.3, the post-interventional expectation of TGL is 110, with a 95% CI of [99, 120]. In contrast, for individuals with CRP = 2, the expectation is 130, with a 95% CI of [110, 150].

#### 3.4.8 HOMA-IR → T2D

Figure 5C shows the average estimate and 95% confidence region for the probabilities of T2D, given different levels of HOMA-IR set by intervention (P(T2D|do(HOMA-IR))) . Since the effect is unconfounded in the PAG, it was adjusted solely using the standard set of covariates. The probability significantly increases with HOMA-IR levels. For instance, for individuals with HOMA-IR = 1, the probability of T2D is 0.02, with a 95% CI of [0.0062, 0.062]. In contrast, for individuals with HOMA-IR = 10.17, it rises to 0.16, with a 95% CI of [0.053, 0.39], and for those with HOMA-IR = 15.25, it further increases to 0.4, with a 95% CI of [0.14, 0.73].

#### 3.4.9 HOMA-IR → TGL

Figure 5D shows the average estimate and 95% confidence region for the expectation of TGL given different levels of HOMA-IR set by intervention (E(TGL|do(HOMA-IR))) . Following the GBDC, unbiased estimates were derived through adjustment by CRP and standard covariates. TGL levels significantly increase with HOMA-IR levels. For individuals with HOMA-IR = 1 the post-interventional expectation of TGL is 100 with a 95% CI of [92, 110], while for individuals with HOMA-IR = 10.17, it is 130 with a 95% CI of [120, 150].

#### 3.4.10 HOMA-IR → HDLc

Figure 5E shows the average estimate and 95% confidence region for the expectation of HDLc given different levels of HOMA-IR set by intervention (E(HDLc|do(HOMA-IR))) . As per GBDC, estimates were derived by adjusting for CRP and standard covariates. HDLc significantly decreases with HOMA-IR levels. For individuals with HOMA-IR = 1 the post-interventional expectation of HDLc is 45 with a 95% CI of [43, 48], while for individuals with HOMA-IR = 10.17, it is 38 with a 95% CI of [35, 41].

#### 3.4.11 TGL → DLP

Figure 5F shows the average estimate and 95% confidence region for the probabilities of DLP, given different levels of TGL set by intervention (P(DLP|do(TLG))) . The probability of DLP significantly increases with TGL levels. Following GBDC, unbiased estimates were derived through adjustment for CRP and the standard set of covariates. For individuals with TGL = 52.2, the post-interventional probability of DLP is 0.54 with a 95% CI of [0.42, 0.65], while for individuals with TGL = 150.6, it is 0.76 with a 95% CI of [0.66, 0.84].

#### 3.4.12 HDLc → DLP

Figure 5G shows the average estimate and 95% confidence region for the probabilities of DLP, given different levels of HDLc set by intervention (P(DLP|do(HDLc))) . Following the GBDC, unbiased estimates were obtained through adjustments for TGL and the standard set of covariates. The probability of DLP is notably elevated for low values of HDLc. With an HDLc of 40.82, the post-interventional probability of DLP reaches 0.89 with a 95% CI of [0.8, 0.94]. As HDLc levels increase, the probability decreases: for HDLc = 50.73, it drops to 0.49, with a 95% CI of [0.34, 0.64], and further decreases to 0.11 for HDLc = 60.64, with a 95% CI of [0.054, 0.2].

## 4 Discussion

Our data-driven approach not only uncovers a causal relationship from obesity to HTN, body inflammation (assessed by CRP levels), and insulin resistance (HOMA-IR), but also provides further evidence that obesity significantly increases the risk of developing these conditions. The analysis also indicates that body inflammation, as indicated by CRP, causally influences insulin resistance, as indicated by HOMA-IR. However, its effect size could not be obtained as statistically significant using our observed sample.

### 4.1 Obesity → HTN

Extensive research HTN established a strong association and a causal link from obesity to HTN, elucidated by complex underlying mechanisms (Jiang et al., 2016; Seravalle and Grassi, 2017; Powell-Wiley et al., 2021). The contribution of obesity to the development and progression of HTN can be attributed to a range of factors. These include the overactivation of the renin-angiotensin-aldosterone system and the sympathetic nervous system, the overstimulation of pro-inflammatory adipokines–such as tumor necrosis factor- α (TNF- α ), leptin, and plasminogen activator inhibitor type 1 –, insulin resistance, immune dysfunction, and structural and functional alterations in renal, cardiac, and adipose tissues (Kotsis et al., 2010; Shams et al., 2022). Unraveling the precise interplay between these factors remains a significant ongoing challenge in the field of medical research.

### 4.2 Obesity → CRP → HOMA-IR → T2D

Numerous studies consistently show that individuals with obesity exhibit higher CRP levels—a marker of low-grade inflammation—compared to those with a healthy weight (Visser et al., 1999; Yudkin et al., 1999; Choi et al., 2013). Obesity primarily contributes to chronic low-grade inflammation by releasing a surge of signaling molecules, including the adipokines leptin and resistin, the cytokine interleukin-6 (IL-6), and the chemokine monocyte chemoattractant protein-1 (MCP-1). These molecules create a pro-inflammatory environment, attracting immune cells called monocytes into adipose tissue. This inflammatory cascade is implicated in obesity-related metabolic dysfunctions, potentially culminating in insulin resistance and the onset of T2D (Chen et al., 2015; Wu and Ballantyne, 2020).

### 4.3 Obesity → HOMA-IR

The contribution of obesity to insulin resistance is not solely mediated through inflammation. Besides inflammation, obesity-driven factors such as elevated levels of free fatty acids (FFA) in muscle and liver tissues can directly disrupt insulin signaling pathways, in a process called lipotoxicity (Ahmed et al., 2021; Filipović et al., 2021). Additionally, adipose tissue dysfunction in obesity leads to the release of proinflammatory cytokines such as IL-6 and TNF- α that can directly interfere with insulin signaling within cells, further exacerbating insulin resistance (Fève and Bastard, 2009).

Our causal analysis reveals that high levels of HOMA-IR significantly contributes not only to the onset of T2D but also to elevated TGL and decreased HDLc levels. Furthermore, in accordance with the definition of DLP, our analysis reveals that both HDLc and TGL causally contribute to DLP, with low levels of HDLc and high levels of TGL significantly increasing the likelihood of DLP.

### 4.4 HOMA-IR → TGL and HDLc → DLP

Various population-based studies have consistently established a correlation between insulin resistance and increased TGL alongside decreased levels of HDLc. For a comprehensive list of references, see, for instance, Howard (1999). Individuals with insulin resistance often exhibit a condition known as diabetic dyslipidemia or dyslipidemia of insulin resistance, marked by elevated TGL levels, decreased levels of HDLc, and normal or slightly elevated levels of LDLc (Bahiru et al., 2021). The potential of a higher TGL/HDLc ratio as a marker for insulin resistance has been explored in several studies (Giannini et al., 2011; Behiry et al., 2019). In insulin resistance, the typical suppression of VLDL production by the liver, a lipoprotein rich in triglycerides, does not occur as expected. Consequently, there is an increase in VLDL production, leading to elevated triglyceride levels in the bloodstream. Additionally, various factors in insulin resistance may contribute to reduced HDL, such as cholesterol ester exchange between HDLc and VLDL triglycerides, increased hepatic lipase activity, and altered hepatic function affecting production of apo AI (the main apoprotein of HDL) and secretion of nascent HDLc (Howard, 1999; Ginsberg et al., 2005).

Certain identified causal relationships exhibited effect sizes that did not reach statistical significance. These included the effects of CRP on HOMA-IR and TGL, as well as the effect of T2D on LDLc. The corresponding effect of the causal relationship from Med_HTN to Med_T2D was also not significant.

### 4.5 CRP → HOMA-IR and TGL

Numerous studies indicate that systemic inflammation significantly influences insulin resistance (Wu and Ballantyne, 2020; Bulcão et al., 2006) and is associated with elevated TGL levels Feingold and Grunfeld (2022); Ronti et al. (2006). The lack of statistical significance in our analysis could be attributed to substantial variability within our dataset, indicating a need for a larger sample size in further investigations.

### 4.6 T2D → LDL

The lack of statistical significance in the effect of T2D on LDLc may indicate that this relationship involves a more complex interplay than a mere quantitative change in LDLc levels. Within our dataset, many diabetic patients have normal or even low LDLc levels, which is consistent with existing studies (Howard et al., 2000). However, T2D can qualitatively alter LDL particles, leading to the formation of smaller, denser particles that are more prone to oxidation and arterial infiltration. These changes increase the risk of atherosclerosis and cardiovascular complications in diabetic individuals (Taskinen, 1992).

### 4.7 Med_HTN → Med_T2D

The causal edge from Med_HTN to Med_T2D appears to be false, given the non-significance of the corresponding effect and the absence of scientific evidence supporting the claim that medication for HTN has a direct effect on medication for T2D. However, some studies have indicated that Med_HTN can increase the risk of T2D Bangalore et al. (2007); Arnold et al. (2020). In this scenario, the causal edge from Med_HTN to Med_T2D may be accounting for an effect that is mediated by T2D but was not accurately represented in the graph. This discrepancy could have occurred because the edge between Med_HTN and T2D may have been mistakenly removed by the algorithm, possibly due to a false identification of conditional independencies from weak correlations. Further exploration is necessary to gain a better understanding of the nature of this relationship and its implications.

## 5 Conclusion, limitations, and final remarks

The conservative RFCI is a classical causal discovery algorithm tailored for scenarios featuring latent confounding. It distinguishes itself by providing superior computational efficiency while maintaining consistency in sparse models, along with enhanced robustness through an approach that avoids tests susceptible to low statistical power (Colombo et al., 2012). However, it tends to produce rather uninformative models when faced with conflicting orientations.

To address this limitation, this paper introduces anchorFCI, a novel adaptation of the conservative RFCI. Given two sets of variables, where the first includes the variables of interest and the second consists solely of those known not to be caused by the first, the algorithm strategically identifies reliable anchor variables and seamlessly integrates their known non-ancestral relationships in the learning process. Reliable anchor variables are defined as those from the second set that are significantly associated with a variable from the first set and, when integrated into the graph, form unambiguous triples based on the majority rule. By effectively identifying and integrating these variables, anchorFCI significantly enhances its capability to orient edges within the conservative framework, thereby increasing both robustness and overall discovery power, particularly in real-world scenarios marked by latent confounding and limited sample sizes.

By employing the anchorFCI, followed by state-of-the-art effect identification tools (Jaber et al., 2022), we conducted a fully data-driven causal analysis of various cardiometabolic risk factors and related SNP variables included in the 2015 ISA-Nutrition dataset (Fisberg et al., 2018). The approach proved effective, uncovering the causal relationships among all the phenotype and clinical variables under consideration. Importantly, many of the identified orientations are strongly supported by existing literature, thereby enhancing the credibility of our findings.

While our investigation has yielded interesting results, it also highlights the critical need for more robust methods for causal discovery from observational data in practical scenarios. A plethora of causal discovery methods have been recently proposed, with many emphasizing scalability (Zheng et al., 2018; Glymour et al., 2019; Vowels et al., 2022). However, they often focus on scenarios that are unrealistic due to the imposition of strong assumptions, including not only faithfulness but also causal sufficiency and various parametric or distributional assumptions. Only a few have been proposed for non-parametric causal discovery in scenarios involving latent confounding (Colombo et al., 2012; Colombo and Maathuis, 2014; Hyttinen et al., 2014; Magliacane et al., 2016). Nevertheless, they still exhibit high vulnerability to violations of the faithfulness assumption, which is commonly observed in real-world settings. Any falsely identified conditional dependence or independence may lead to findings that deviate from the truth and our current understanding. Hence, there is an urgent demand for methods that demonstrate greater resilience in situations with limited data, capable of adeptly managing conflicting orientations, accommodating uncertainty in the learning process, and integrating background knowledge.

## Data Availability

The data analyzed in this study is subject to the following licenses/restrictions: The dataset utilized in this study is not publicly available. Requests to access these datasets should be directed to Regina Mara Fisberg. Further details can be found at www.gac-usp.com.br.
