# Supplementary material for: AnchorFCI: harnessing genetic anchors for enhanced causal discovery of cardiometabolic disease pathways
Source: Front Genet. 2024 Dec 9;15:1436947. doi: 10.3389/fgene.2024.1436947 (PMC11663939; doi:10.3389/fgene.2024.1436947)
Supplement: Supplementary file 1 [file DataSheet1.pdf]

Supplementary Material

1 COMPARATIVE STUDY BETWEEN RFCI AND ANCHOR FCI

Table S1. Comparison of RFCI and anchorFCI performance in learning the structure among the variables of interest and all three potential anchors. Scores represent the difference between the SHD of the inferred PAG relative to the true MAG and the SHD of the true PAG relative to the true MAG. Values are averages across 50 randomly generated MAGs, each evaluated on 30 datasets with varying sample sizes N. #Anchors indicates the average number of selected reliable anchors by anchorFCI. Smaller scores indicate higher accuracy, with zero indicating performance equivalent to the true PAG and negative scores suggesting greater informativeness beyond the MEC. P-values are from a one-sided Wilcoxon test with alternative hypothesis that the average difference between RFCI and anchorFCI scores (Diff) is greater than 0.

N	RFCI		AnchorFCI		Diff	P-Value
	[Min, Max]	Mean ± SD	[Min, Max]	Mean ± SD		
500	[3.03, 9.43]	7.04 ± 1.46	[-0.57, 5.67]	3.49 ± 1.39	3.55	4.94×10^{-248}
1000	[2.13, 7.17]	5.64 ± 1.30	[-1.33, 4.10]	2.42 ± 1.32	3.22	5.43×10^{-248}
5000	[1.37, 4.97]	3.60 ± 0.96	[-1.97, 2.87]	0.77 ± 1.15	2.83	3.58×10^{-251}
10000	[1.37, 4.50]	3.07 ± 0.81	[-2.50, 1.67]	0.29 ± 1.01	2.78	3.61×10^{-252}

2 CAUSAL DISCOVERY USING THE CONSERVATIVE RFCI ALGORITHM

Figure S1 shows the Partial Ancestral Graph (PAG) obtained by employing the conservative RFCI algorithm with majority rule, excluding any genetic variables from consideration.

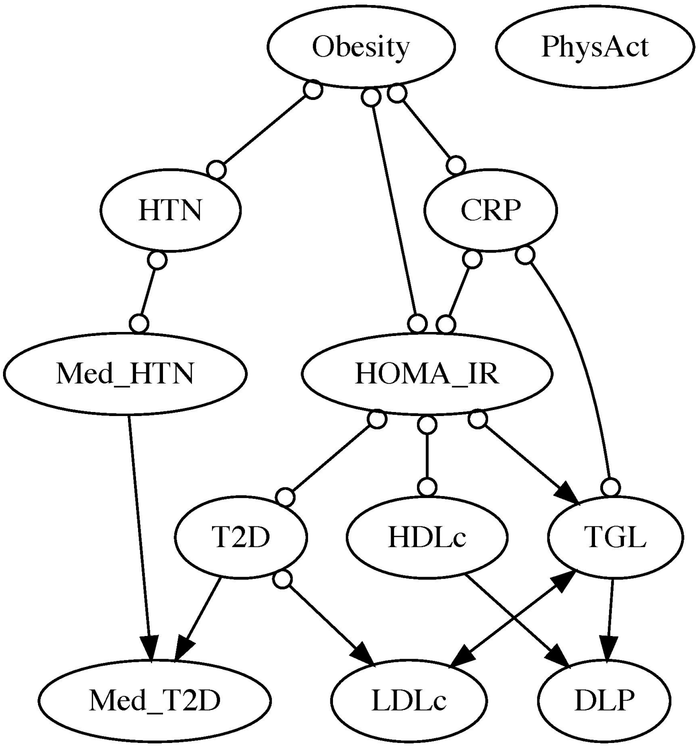

Figure S1. Partial Ancestral Graph inferred by the conservative RFCI algorithm with majority rule, *without using any SNP variables*. Directed edges imply ancestral (causal) relationships. Bidirected edges imply purely spurious associations. Circles indicate uncertain edge-marks, interchangeable with tail or arrowhead in equally probable models. Nodes representing various phenotypic traits include: Obesity, Type 2 Diabetes (T2D), Hypertension (HTN), Dyslipidemia (DLP), C-reactive protein (CRP), Homeostatic Model Assessment of Insulin Resistance (HOMA-IR), Triglycerides (TGL), Low-Density Lipoprotein Cholesterol (LDLc), High-Density Lipoprotein Cholesterol (HDLc), Medication for T2D (Med_T2D), Medication for HTN (Med_HTN), and Physical Activity (PhysAct).

Figure S2 shows the Partial Ancestral Graph (PAG) obtained by employing the conservative RFCI algorithm with majority rule, including the set of genetic variables from consideration.

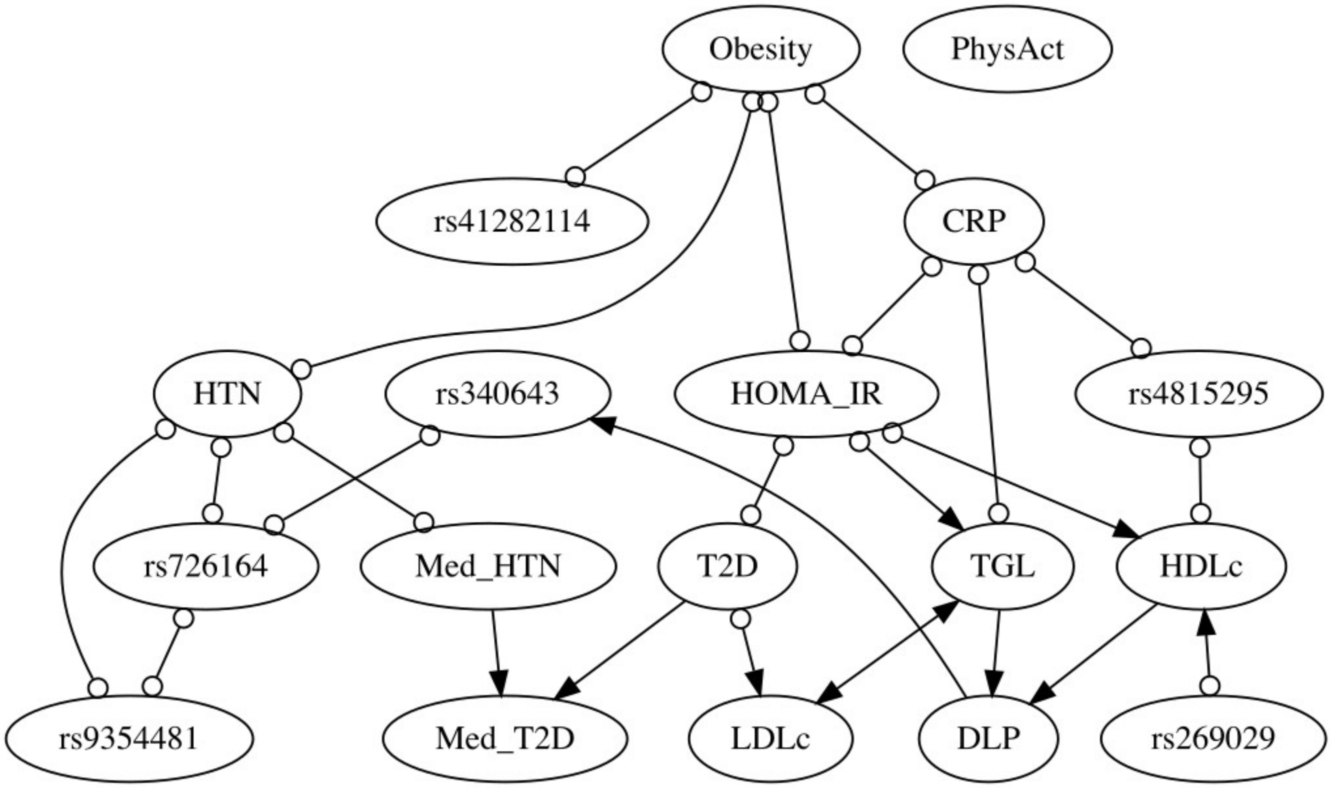

Figure S2. Partial Ancestral Graph inferred by the conservative RFCI algorithm with majority rule, *including SNP variables* are part of the general set of observed variables. Directed edges imply ancestral (causal) relationships. Bidirected edges imply purely spurious associations. Circles indicate uncertain edge-marks, interchangeable with tail or arrowhead in equally probable models. Nodes representing various phenotypic traits include: Obesity, Type 2 Diabetes (T2D), Hypertension (HTN), Dyslipidemia (DLP), C-reactive protein (CRP), Homeostatic Model Assessment of Insulin Resistance (HOMA-IR), Triglycerides (TGL), Low-Density Lipoprotein Cholesterol (LDLc), High-Density Lipoprotein Cholesterol (HDLc), Medication for T2D (Med_T2D), Medication for HTN (Med_HTN), and Physical Activity (PhysAct).

3 BOOSTRAPPED ANCHOR FCI RESULTS

Table S2. Percentages of inferred edge types across 50 bootstrap samples utilizing all considered phenotypes and genotypes. The percentages corresponding to the edge type inferred from the original dataset are highlighted in bold.

Relationship	∅	○–○	←○	–○	○→	↔	→	○–	←	–
Obesity - HTN	38	16	6	0	2	0	**24**	0	14	0
Obesity - CRP	4	2	8	0	2	16	**46**	0	22	0
Obesity - HOMA-IR	0	0	0	0	0	4	**88**	0	8	0
T2D - HOMA-IR	0	0	0	0	12	10	24	0	**54**	0
T2D - LDLc	14	6	4	0	16	12	**44**	0	4	0
T2D - Med T2D	0	2	0	0	20	42	**36**	0	0	0
HTN - Med HTN	0	2	4	0	0	2	**80**	0	12	0
DLP - HDLc	0	0	0	0	0	14	28	0	**58**	0
DLP - TGL	0	0	0	0	0	6	10	0	**84**	0
CRP - HOMA-IR	38	0	0	0	0	8	**42**	0	12	0
CRP - TGL	52	0	0	0	0	10	**16**	0	22	0
HOMA-IR - HDLc	42	0	0	0	0	20	**26**	0	12	0
HOMA-IR - TGL	0	0	0	0	0	50	**50**	0	0	0
LDLc - TGL	0	0	0	0	16	**64**	16	0	4	0
Med T2D - Med HTN	0	0	4	0	0	36	2	0	**58**	0
Obesity - rs41282114	8	0	86	0	0	6	0	0	0	0
HTN - rs726164	0	0	40	0	0	54	0	0	6	0
HTN - rs9354481	14	0	54	0	0	30	0	0	2	0
DLP - rs340643	8	0	32	0	0	52	0	0	8	0
CRP - rs4815295	38	0	26	0	0	28	0	0	8	0
HDLc - rs269029	2	0	42	0	0	52	0	0	4	0
HDLc - rs4815295	4	0	44	0	0	42	0	0	10	0
PhysAct - rs4815295	64	0	28	0	0	6	0	0	2	0
rs726164 - rs9354481	36	32	10	0	4	6	10	0	2	0
rs726164 - rs340643	28	14	14	0	10	12	8	0	14	0

Table S3. Percentages of inferred edge types across 50 bootstrap samples utilizing a small subset of the considered phenotypes and genotypes. The percentages corresponding to the edge type inferred from the original dataset are highlighted in bold.

Relationship	∅	o–o	←o	–o	o→	↔	→	o–	←	–
Obesity - HOMA-IR	0	0	0	0	0	0	**100**	0	0	0
T2D - HOMA-IR	0	0	0	0	10	0	8	0	**82**	0
T2D - LDLc	0	10	0	0	6	2	**82**	0	0	0
DLP - HDLc	0	0	0	0	0	6	40	0	**54**	0
DLP - TGL	0	0	0	0	4	2	4	0	**90**	0
HOMA-IR - HDLc	38	0	4	0	0	12	**38**	0	8	0
HOMA-IR - TGL	0	0	0	0	0	22	**78**	0	0	0
LDLc - TGL	0	0	0	0	18	**54**	26	0	2	0
Obesity - rs41282114	0	0	**98**	0	0	2	0	0	0	0
HDLc - rs4815295	2	0	**76**	0	0	20	0	0	2	0

4 SUPPLEMENTARY TABLES

Table S4. Coefficient estimates, their corresponding statistics, and p-values from a logistic regression for hypertension, accounting for standard covariates (sex, age, age^2, PC1, and PC2) and Obesity.

	Estimate	Std. Error	z value	Pr(>|z|)
(Intercept)	-2.2	0.52	-4.3	1.7×10^{-5}
sex	-0.058	0.19	-0.31	0.75
age	0.032	0.021	1.5	0.13
age^2	0.00025	0.00024	1	0.3
PC1	26	21	1.2	0.22
PC2	16	27	0.61	0.54
Obesity	1.1	0.22	4.9	7.8×10^{-7}

Table S5. Coefficient estimates, their corresponding statistics, and p-values from a normal linear regression for the log-transformed levels of CRP, accounting for standard covariates (sex, age, age^2, PC1, and PC2) and Obesity.

	Estimate	Std. Error	t value	Pr(>|t|)
(Intercept)	-3.1	0.21	-15	6.5×10^{-42}
sex	0.16	0.083	2	0.05
age	0.05	0.009	5.5	4.7×10^{-8}
age^2	-0.00036	0.0001	-3.6	0.00029
PC1	10	9	1.1	0.27
PC2	-27	12	-2.3	0.021
Obesity	0.6	0.099	6.1	2×10^{-9}

Table S6. Coefficient estimates, their corresponding statistics, and p-values from a normal linear regression for the log-transformed levels of HOMA-IR, accounting for standard covariates (sex, age, age^2, PC1, and PC2) and Obesity.

	Estimate	Std. Error	t value	Pr(>|t|)
(Intercept)	1	0.13	8	4×10^{-15}
sex	0.013	0.049	0.27	0.79
age	-0.006	0.0053	-1.1	0.26
age^2	7.9×10^{-5}	5.9×10^{-5}	1.3	0.18
PC1	-5.9	5.4	-1.1	0.28
PC2	9.6	6.9	1.4	0.16
Obesity	0.72	0.059	12	4.2×10^{-31}

Table S7. Coefficient estimates, their corresponding statistics, and p-values from a normal linear regression for the log-transformed levels of LDLc, accounting for standard covariates (sex, age, age^2, PC1, and PC2) and T2D.

	Estimate	Std. Error	t value	Pr(>|t|)
(Intercept)	4.1	0.071	58	3.3×10^{-262}
sex	0.083	0.027	3.1	0.0022
age	0.023	0.003	7.8	1.9×10^{-14}
age^2	-0.0002	3.3×10^{-5}	-6	4.1×10^{-9}
PC1	-2	3	-0.67	0.5
PC2	3.7	3.8	0.97	0.33
T2D	-0.11	0.041	-2.6	0.0084

Table S8. Coefficient estimates, their corresponding statistics, and p-values from a logistic regression for Med_T2D, accounting for standard covariates (sex, age, age^2, PC1, and PC2), the confounder Obesity, and T2D.

	Estimate	Std. Error	z value	Pr(>|z|)
(Intercept)	-20	1.1×10^3	-0.018	0.99
sex	0.78	0.46	1.7	0.092
age	-0.12	0.17	-0.69	0.49
age^2	0.00077	0.0014	0.57	0.57
PC1	94	71	1.3	0.19
PC2	-76	83	-0.91	0.36
T2D	24	1.1×10^3	0.022	0.98
Obesity	-0.11	0.47	-0.24	0.81

Table S9. Coefficient estimates, their corresponding statistics, and p-values from a logistic regression for Med_HTN, accounting for standard covariates (sex, age, age^2, PC1, and PC2) and HTN.

	Estimate	Std. Error	z value	Pr(>|z|)
(Intercept)	-26	8.3×10^2	-0.032	0.98
sex	0.93	0.26	3.6	0.00032
age	0.2	0.046	4.3	1.6×10^{-5}
age^2	-0.0015	0.0004	-3.7	0.00024
PC1	-26	36	-0.73	0.47
PC2	22	46	0.48	0.63
HTN	20	8.3×10^2	0.024	0.98

Table S10. Coefficient estimates, their corresponding statistics, and p-values from a logistic regression for Med_DLP, accounting for standard covariates (sex, age, age^2, PC1, and PC2) and DLP.

	Estimate	Std. Error	z value	Pr(>|z|)
(Intercept)	-34	1×10^3	-0.034	0.97
sex	0.53	0.31	1.7	0.086
age	0.37	0.17	2.2	0.029
age^2	-0.0024	0.0013	-1.8	0.064
PC1	-31	48	-0.64	0.52
PC2	-14	56	-0.26	0.8
DLP	18	1×10^3	0.018	0.99

Table S11. Coefficient estimates, their corresponding statistics, and p-values from a normal linear regression for log-transformed levels of HOMA-IR, accounting for standard covariates (sex, age, age^2, PC1, and PC2), the confounder Obesity, and CRP.

	Estimate	Std. Error	t value	Pr(>|t|)
(Intercept)	1	0.13	8.1	2.2×10^{-15}
sex	0.005	0.049	0.1	0.92
age	-0.0076	0.0053	-1.4	0.15
age^2	8.8×10^{-5}	5.9×10^{-5}	1.5	0.14
PC1	-6.5	5.4	-1.2	0.22
PC2	11	6.8	1.6	0.11
CRP	0.14	0.044	3.1	0.0022
Obesity	0.68	0.06	11	8×10^{-28}

Table S12. Coefficient estimates, their corresponding statistics, and p-values from a normal linear regression for log-transformed levels of TGL, accounting for standard covariates (sex, age, age^2, PC1, and PC2), the confounder Obesity, and CRP.

	Estimate	Std. Error	t value	Pr(>|t|)
(Intercept)	4.1	0.093	44	1.8×10^{-200}
sex	-0.11	0.036	-3.1	0.002
age	0.029	0.0039	7.3	7.6×10^{-13}
age^2	-0.00026	4.3×10^{-5}	-5.9	6.1×10^{-9}
PC1	-13	3.9	-3.3	0.00094
PC2	9.6	5	1.9	0.056
CRP	0.1	0.032	3.2	0.0012
Obesity	0.1	0.044	2.4	0.018

Table S13. Coefficient estimates, their corresponding statistics, and p-values from a logistic regression model for Type 2 Diabetes, accounting for standard covariates (sex, age, age^2, PC1, and PC2) and HOMA-IR.

	Estimate	Std. Error	z value	Pr(>|z|)
(Intercept)	-11	2	-5.3	1×10^{-7}
sex	0.3	0.25	1.2	0.23
age	0.24	0.065	3.8	0.00018
age^2	-0.0016	0.00054	-3	0.0022
PC1	-7.4	33	-0.22	0.82
PC2	21	42	0.5	0.62
HOMA_IR	0.24	0.037	6.5	6.4×10^{-11}

Table S14. Coefficient estimates, their corresponding statistics, and p-values from a normal linear regression for the log-transformed levels of HDLc, accounting for standard covariates (sex, age, age^2, PC1, and PC2), the confounder CRP, and HOMA-IR.

	Estimate	Std. Error	t value	Pr(>|t|)
(Intercept)	3.8	0.062	62	1.5×10^{-278}
sex	0.072	0.024	3	0.0024
age	-0.0057	0.0026	-2.2	0.028
age^2	7.1×10^{-5}	2.9×10^{-5}	2.5	0.013
PC1	3.5	2.6	1.4	0.18
PC2	-4.8	3.3	-1.4	0.15
HOMA_IR	-0.015	0.0036	-4.2	2.8×10^{-5}
CRP	-0.067	0.022	-3.1	0.002

Table S15. Coefficient estimates, their corresponding statistics, and p-values from a normal linear regression for the log-transformed levels of TGL, accounting for standard covariates (sex, age, age^2, PC1, and PC2), the confounder CRP, and HOMA-IR.

	Estimate	Std. Error	t value	Pr(>|t|)
(Intercept)	4	0.091	44	9.5×10^{-200}
sex	-0.11	0.035	-3	0.0024
age	0.028	0.0038	7.4	2.9×10^{-13}
age^2	-0.00025	4.2×10^{-5}	-6	3.4×10^{-9}
PC1	-12	3.8	-3.2	0.0013
PC2	8.3	4.9	1.7	0.087
HOMA_IR	0.037	0.0053	6.9	9×10^{-12}
CRP	0.079	0.031	2.5	0.012

Table S16. Coefficient estimates, their corresponding statistics, and p-values from a logistic regression model for DLP, accounting for standard covariates (sex, age, age^2, PC1, and PC2), the confounder TGL, and HLDc.

	Estimate	Std. Error	z value	Pr(>|z|)
(Intercept)	7.5	0.91	8.2	3.5×10^{-16}
sex	0.97	0.26	3.8	0.00014
age	0.064	0.027	2.4	0.017
age^2	-0.00049	0.0003	-1.6	0.1
PC1	30	25	1.2	0.24
PC2	-44	32	-1.4	0.17
HLDc	-0.21	0.018	-12	2.4×10^{-33}
TGL	0.0048	0.002	2.4	0.014

Table S17. Coefficient estimates, their corresponding statistics, and p-values from a logistic regression model for DLP, accounting for standard covariates (sex, age, age^2, PC1, and PC2), the confounder CRP, and TGL.

	Estimate	Std. Error	z value	Pr(>|z|)
(Intercept)	-1.3	0.45	-3	0.0028
sex	0.14	0.17	0.82	0.41
age	0.029	0.019	1.5	0.13
age^2	-0.00029	0.00021	-1.4	0.16
PC1	8.7	18	0.48	0.63
PC2	-9	23	-0.38	0.7
TGL	0.01	0.002	5	4.8×10^{-7}
CRP	0.45	0.17	2.6	0.0083

Table S18. Coefficient estimates, their corresponding statistics, and p-values from a logistic regression model for Med_T2D, accounting for standard covariates (sex, age, age^2, PC1, and PC2), the confounder Obesity, and Med_HTN.

	Estimate	Std. Error	z value	Pr(>|z|)
(Intercept)	-7.9	1.8	-4.4	1.3×10^{-5}
sex	0.14	0.29	0.49	0.62
age	0.15	0.06	2.5	0.012
age^2	-0.0011	0.00052	-2.2	0.027
PC1	12	39	0.32	0.75
PC2	-3.8	50	-0.078	0.94
med_HTN	2.1	0.35	6.1	1.1×10^{-9}
Obesity	0.16	0.31	0.51	0.61
