# Supplementary figures and images for: AnchorFCI: harnessing genetic anchors for enhanced causal discovery of cardiometabolic disease pathways
Source: Front Genet. 2024 Dec 9;15:1436947. doi: 10.3389/fgene.2024.1436947 (PMC11663939; doi:10.3389/fgene.2024.1436947)

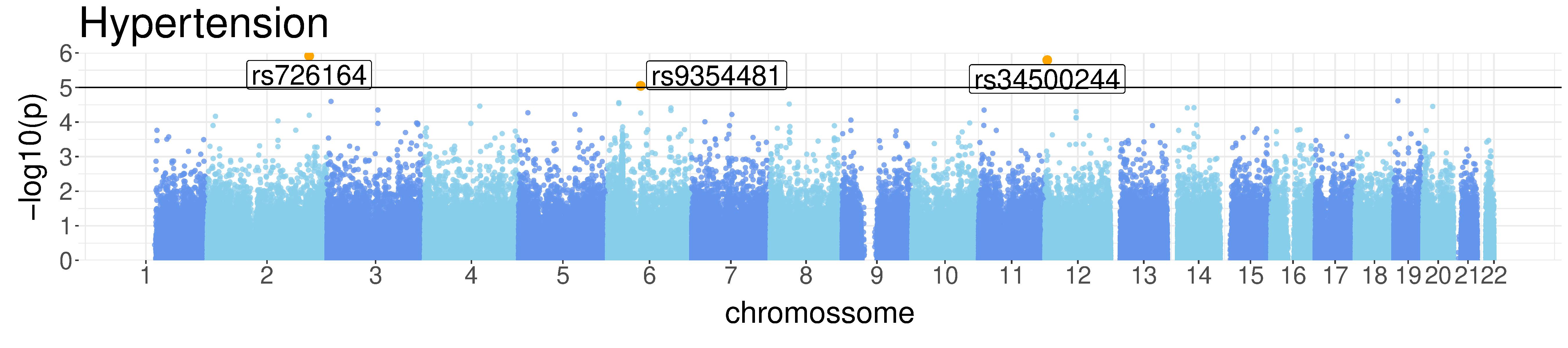

Supplement: Supplementary file 2 [file DataSheet2.zip › figures/Fig1B.jpg]

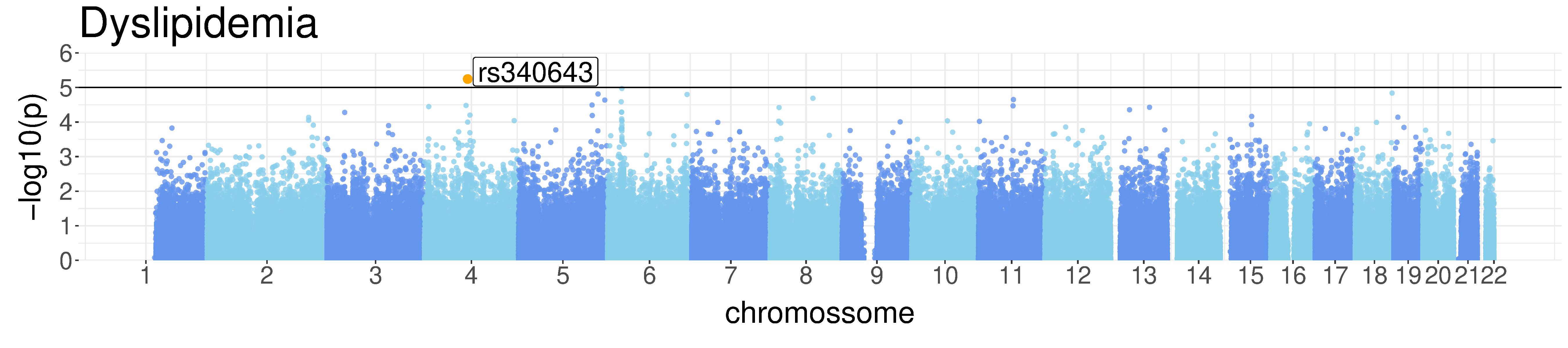

Supplement: Supplementary file 2 [file DataSheet2.zip › figures/Fig1C.jpg]

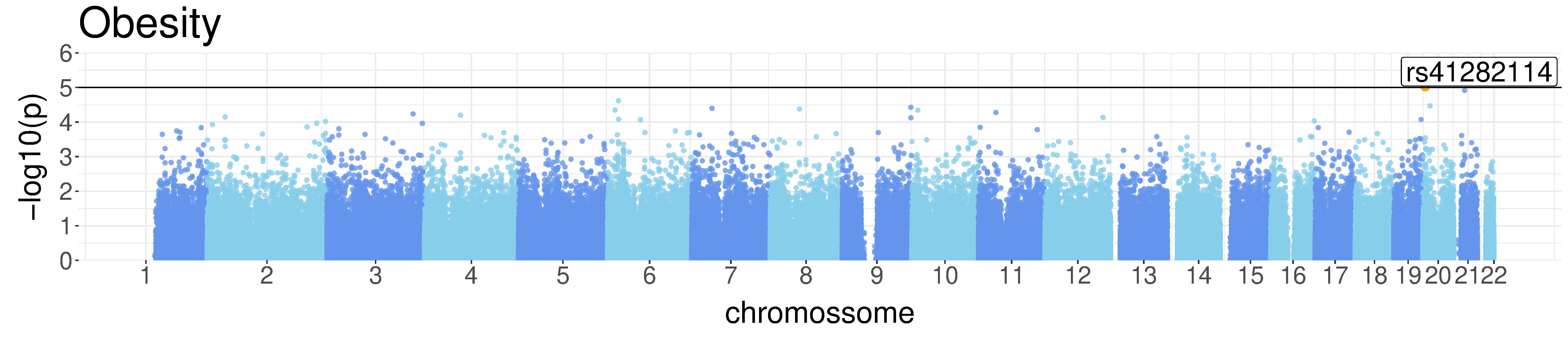

Supplement: Supplementary file 2 [file DataSheet2.zip › figures/Fig1A.jpg]

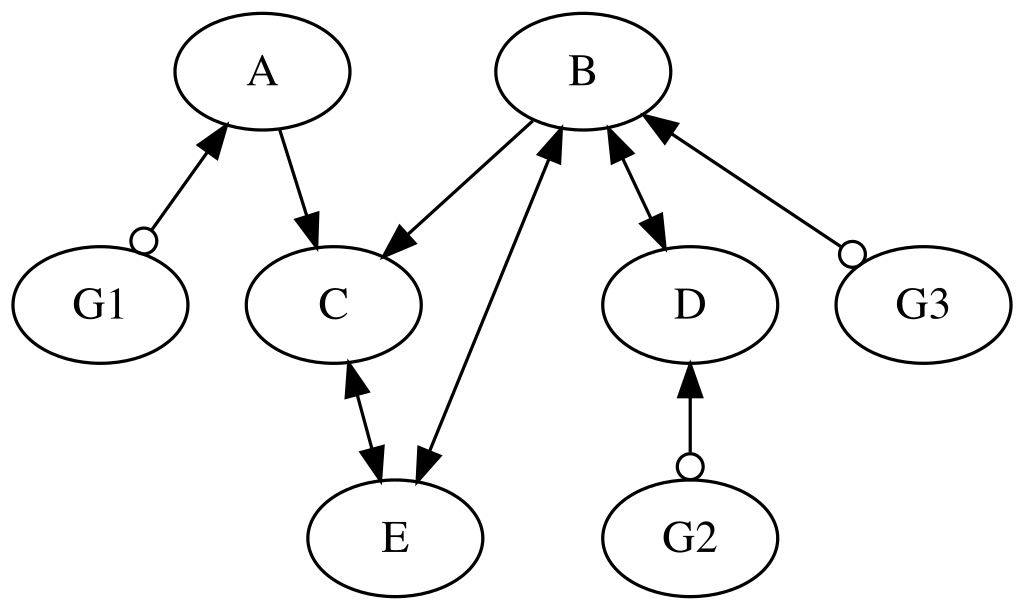

Supplement: Supplementary file 2 [file DataSheet2.zip › figures/Fig0D.jpg]

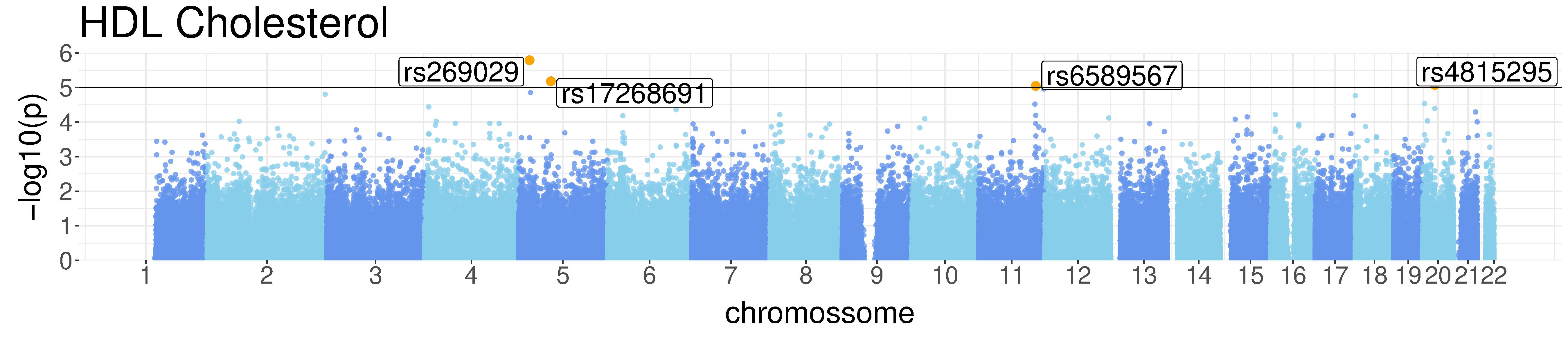

Supplement: Supplementary file 2 [file DataSheet2.zip › figures/Fig1D.jpg]

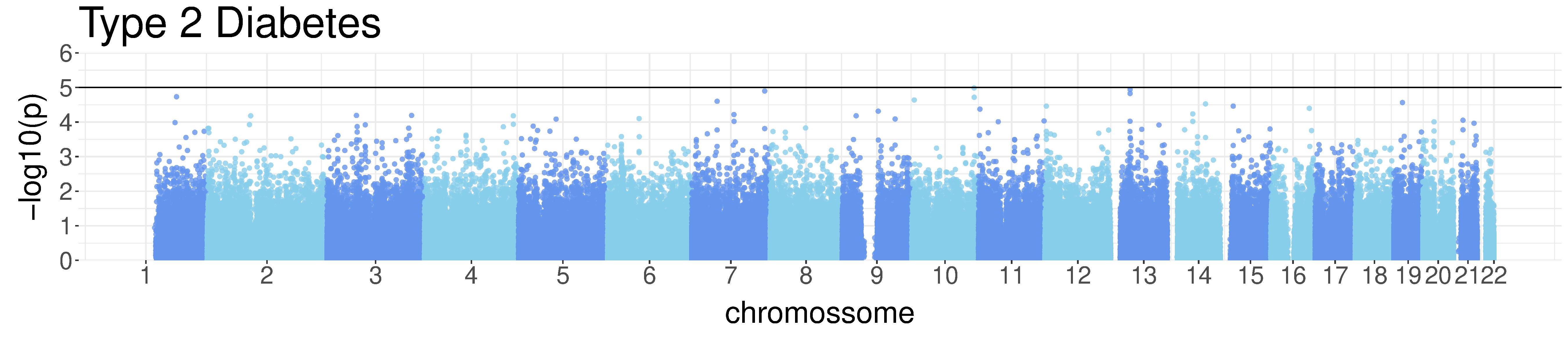

Supplement: Supplementary file 2 [file DataSheet2.zip › figures/Fig1E.jpg]

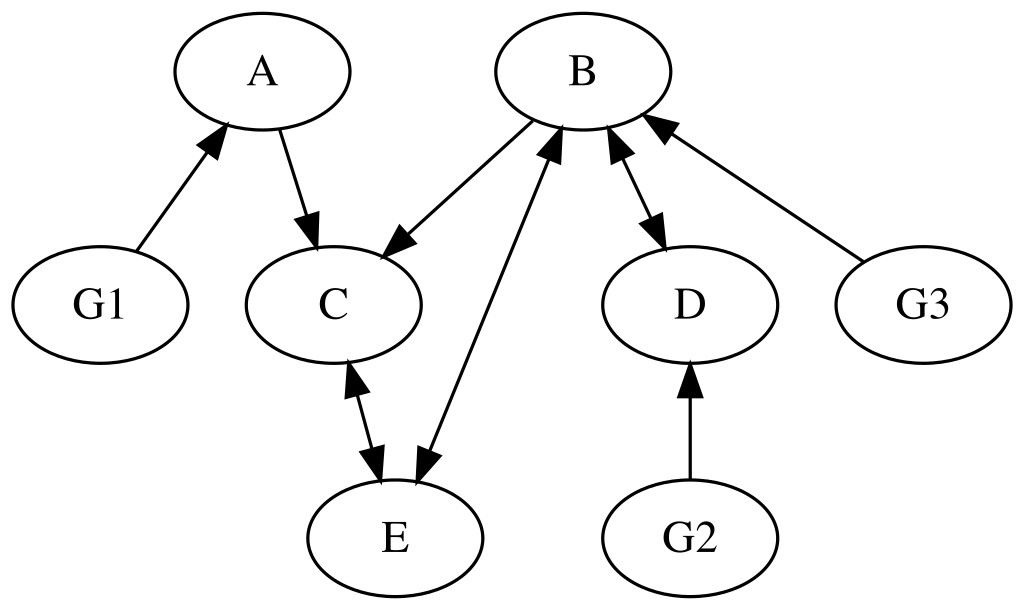

Supplement: Supplementary file 2 [file DataSheet2.zip › figures/Fig0A.jpg]

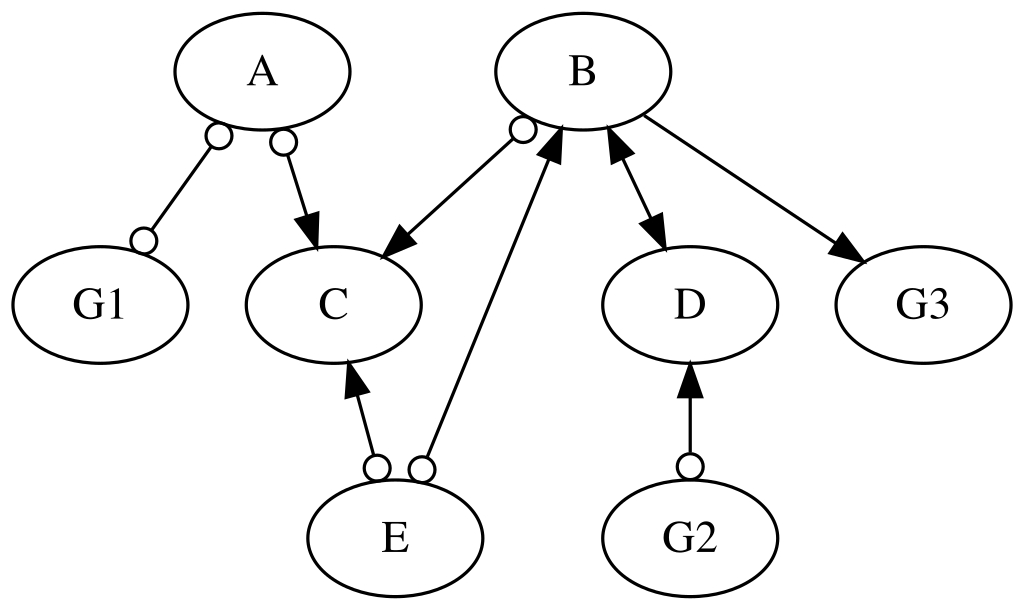

Supplement: Supplementary file 2 [file DataSheet2.zip › figures/Fig0C.jpg]

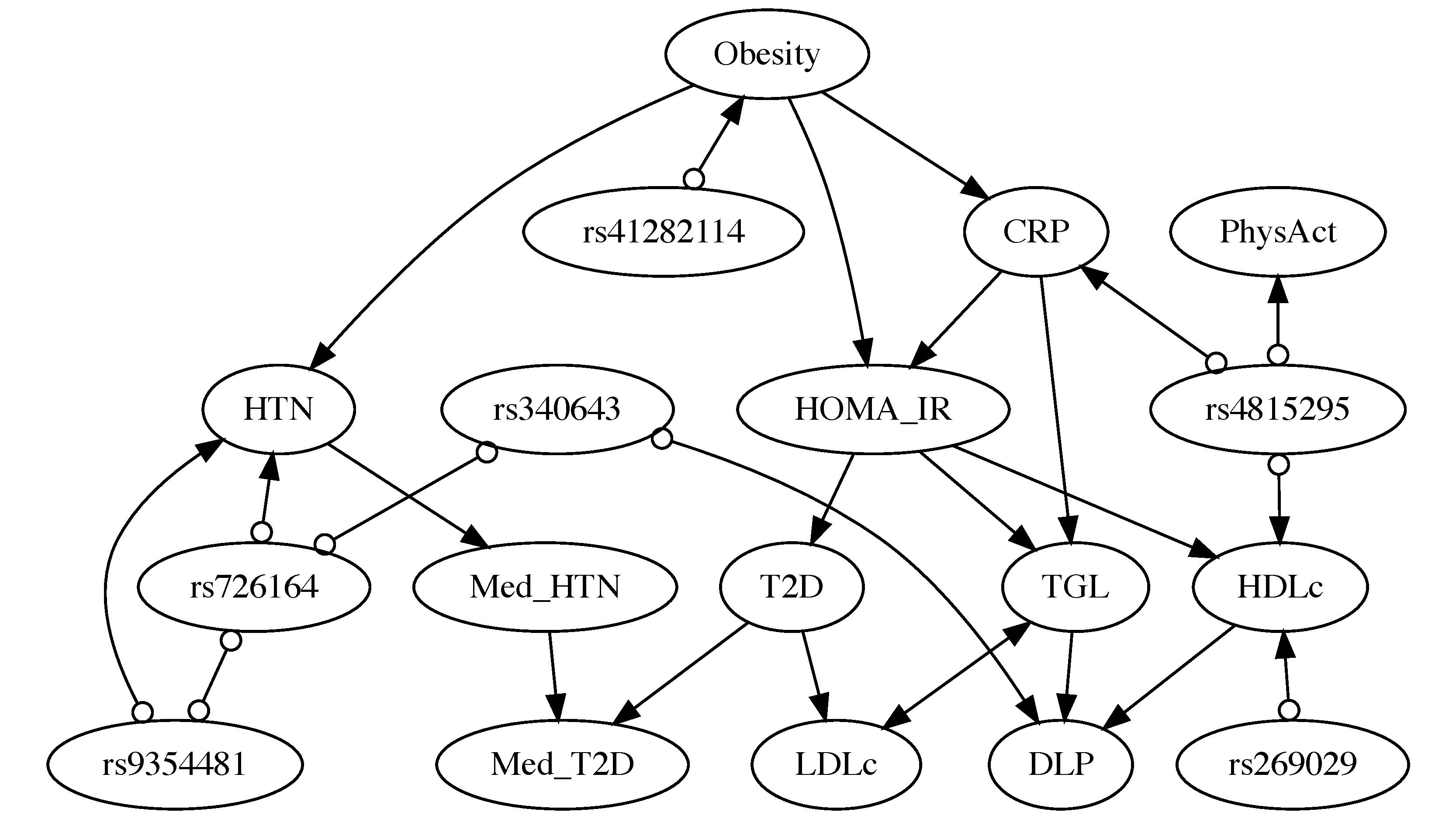

Supplement: Supplementary file 2 [file DataSheet2.zip › figures/FIg2.jpg]

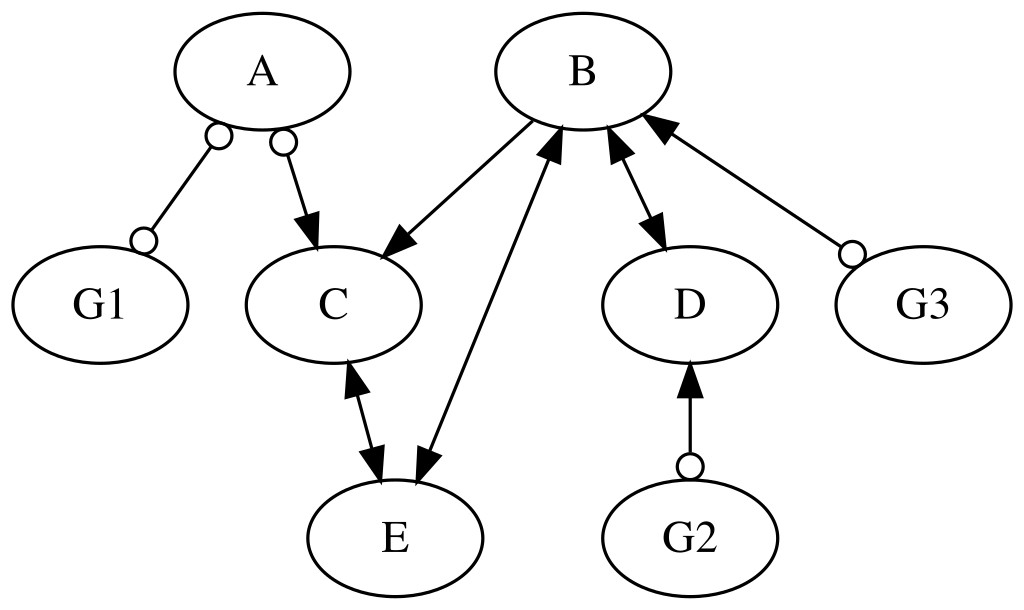

Supplement: Supplementary file 2 [file DataSheet2.zip › figures/Fig0B.jpg]

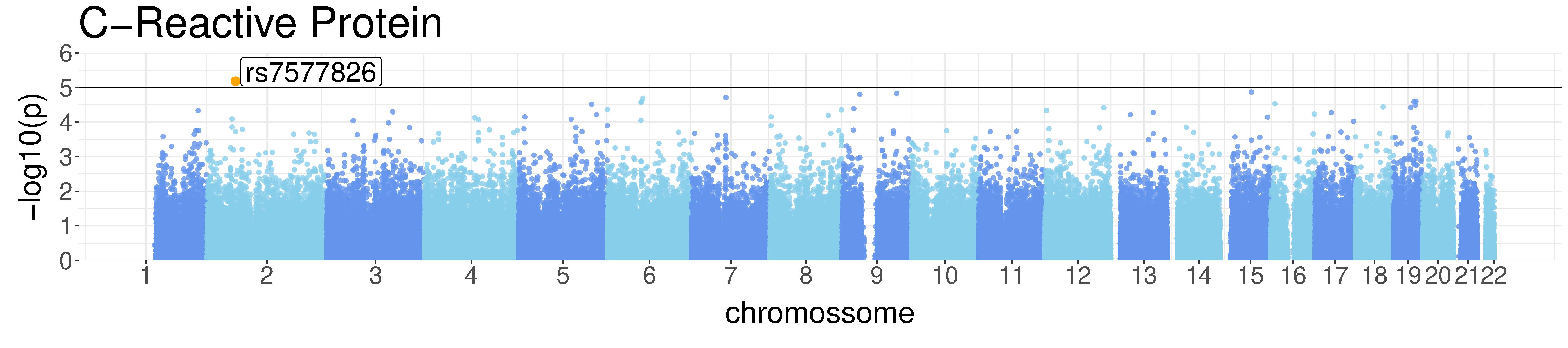

Supplement: Supplementary file 2 [file DataSheet2.zip › figures/Fig1F.jpg]

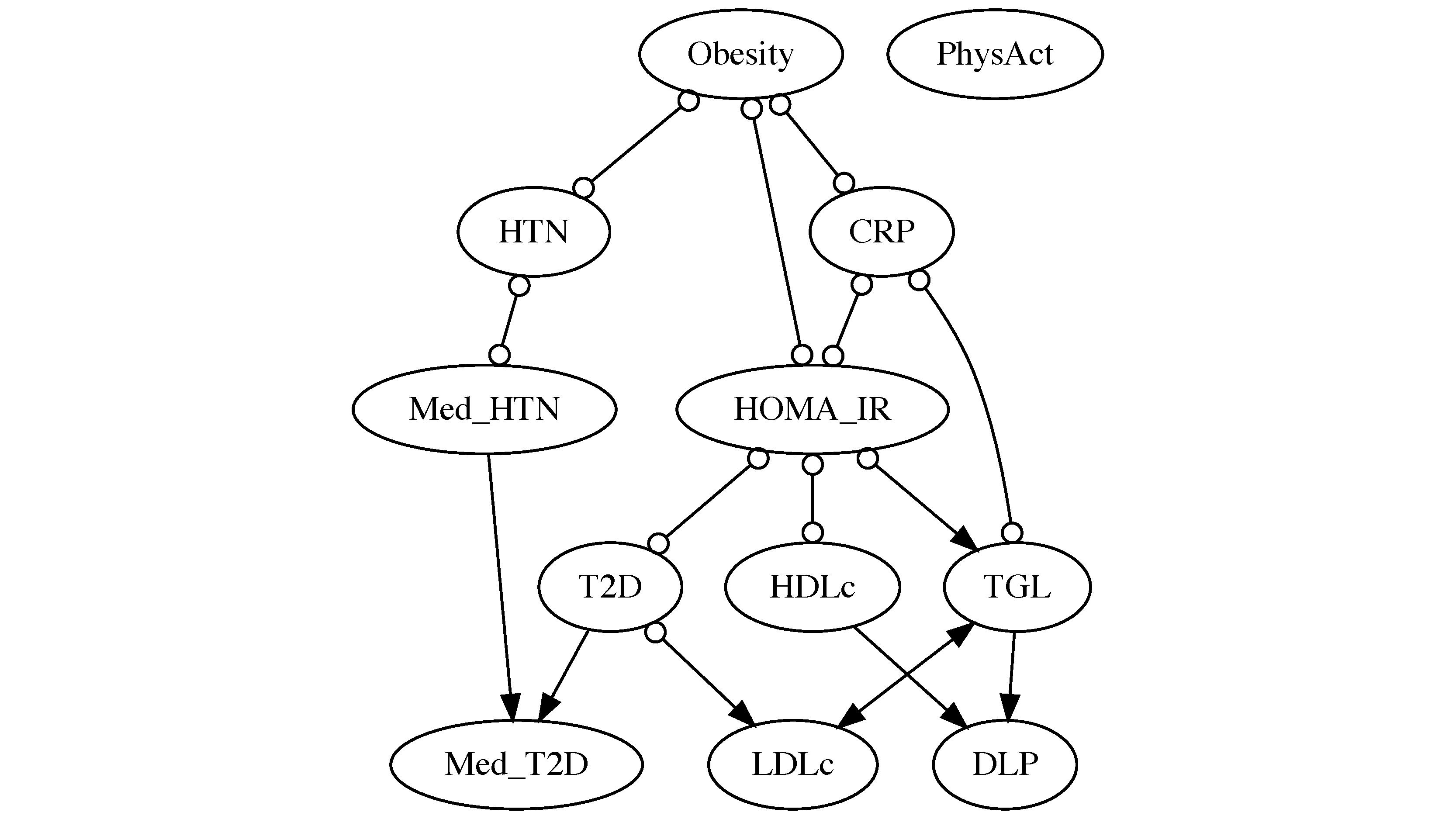

Supplement: Supplementary file 2 [file DataSheet2.zip › figures/FigSuppl1.jpg]

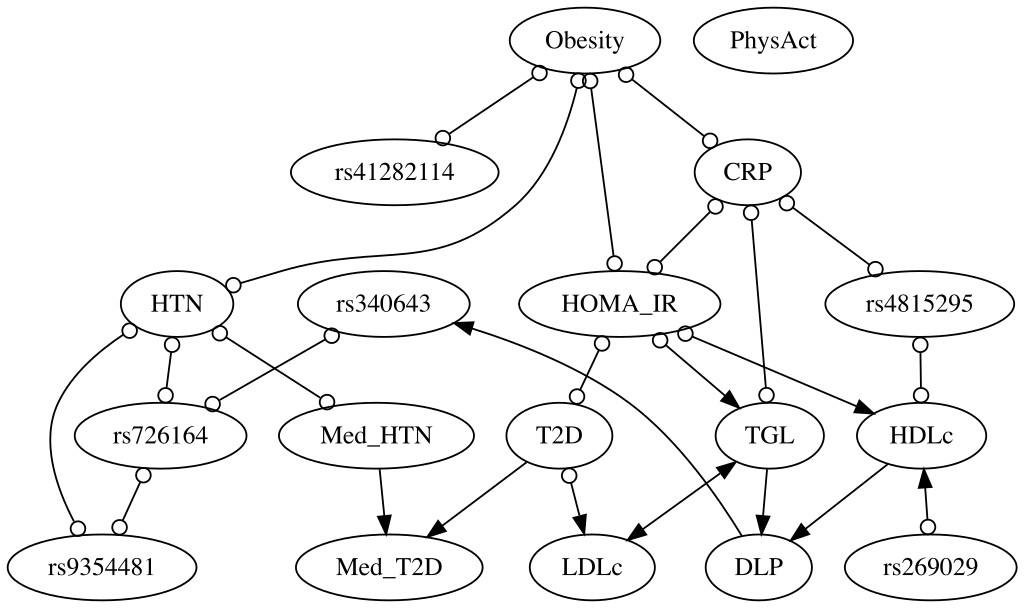

Supplement: Supplementary file 2 [file DataSheet2.zip › figures/FigSuppl2.jpg]

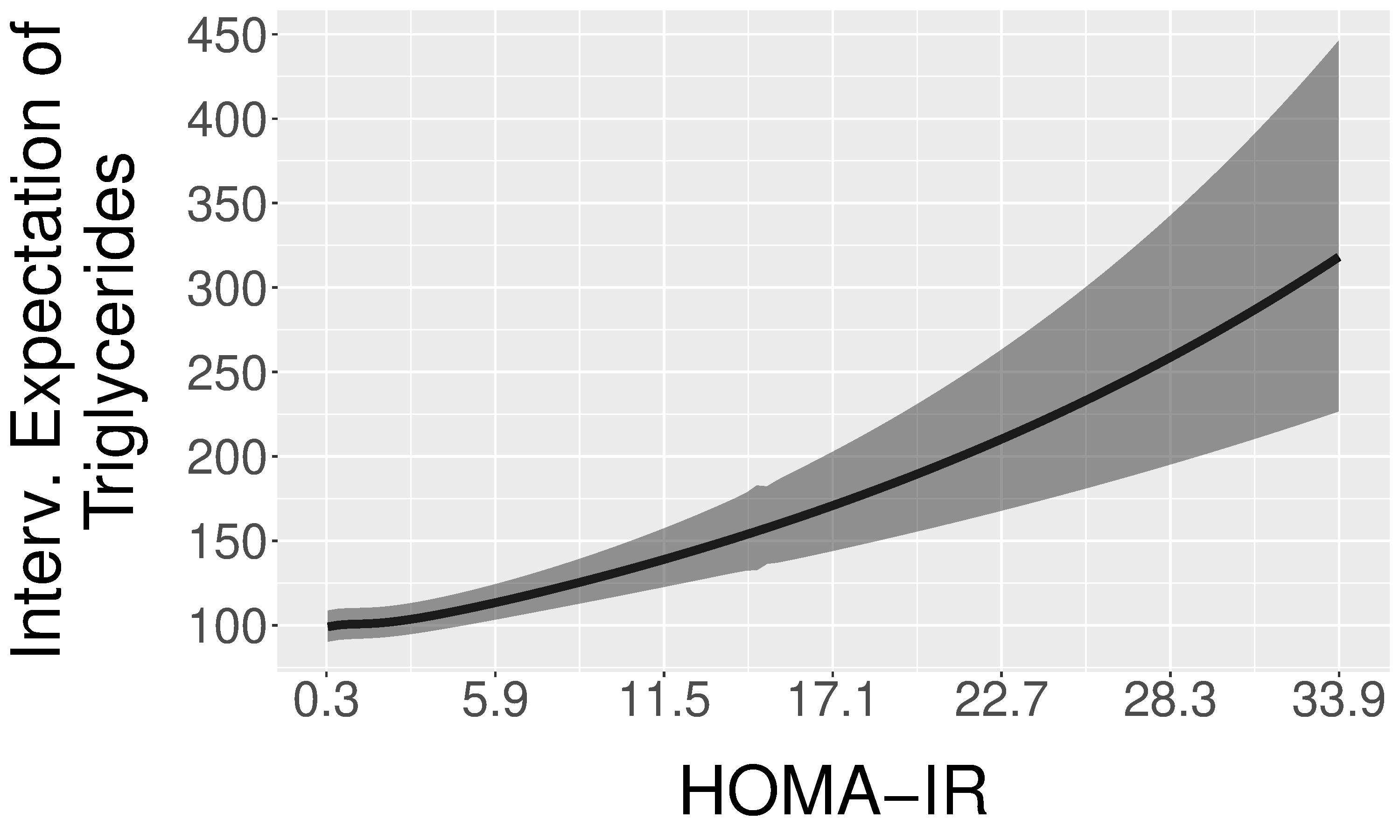

Supplement: Supplementary file 2 [file DataSheet2.zip › figures/Fig4D.jpg]

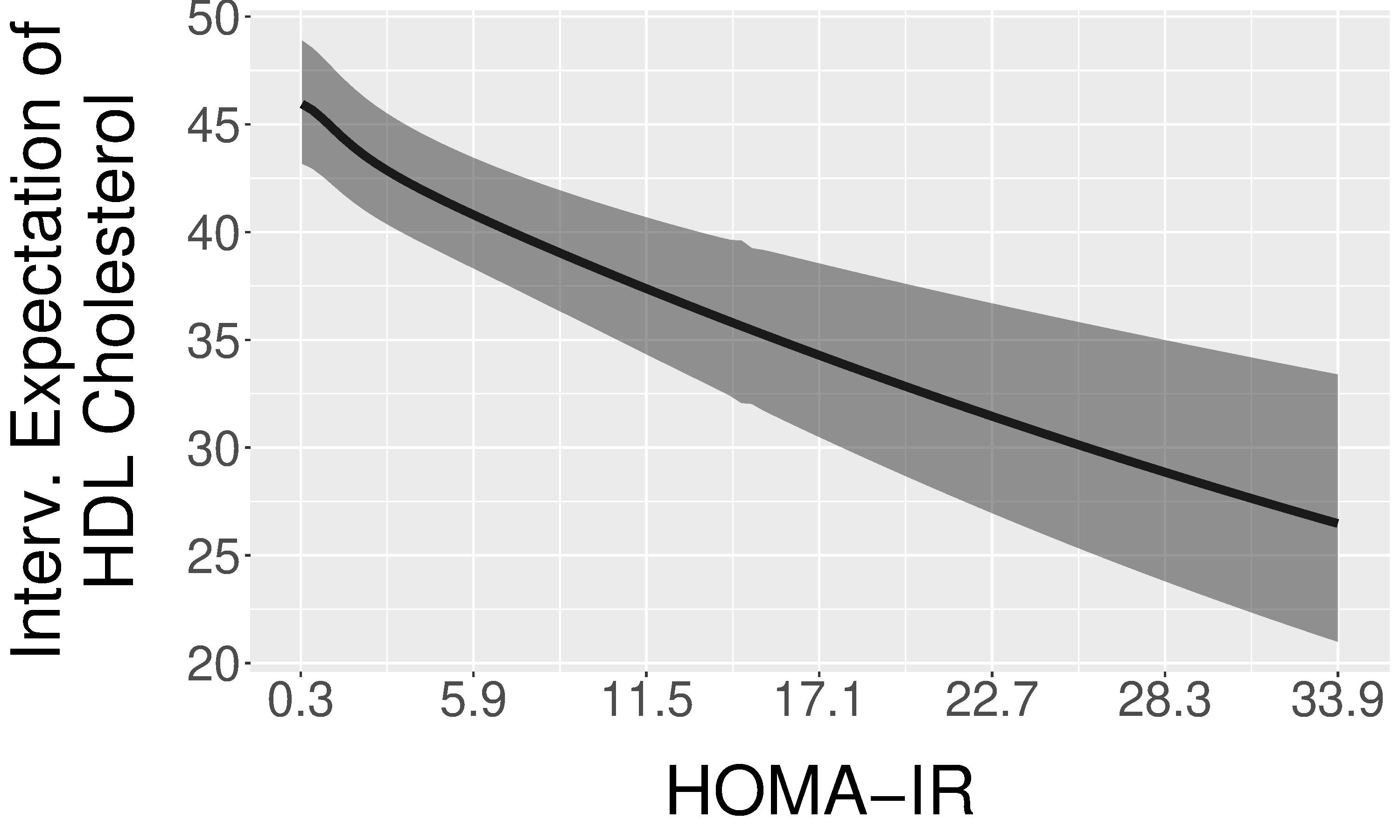

Supplement: Supplementary file 2 [file DataSheet2.zip › figures/Fig4E.jpg]

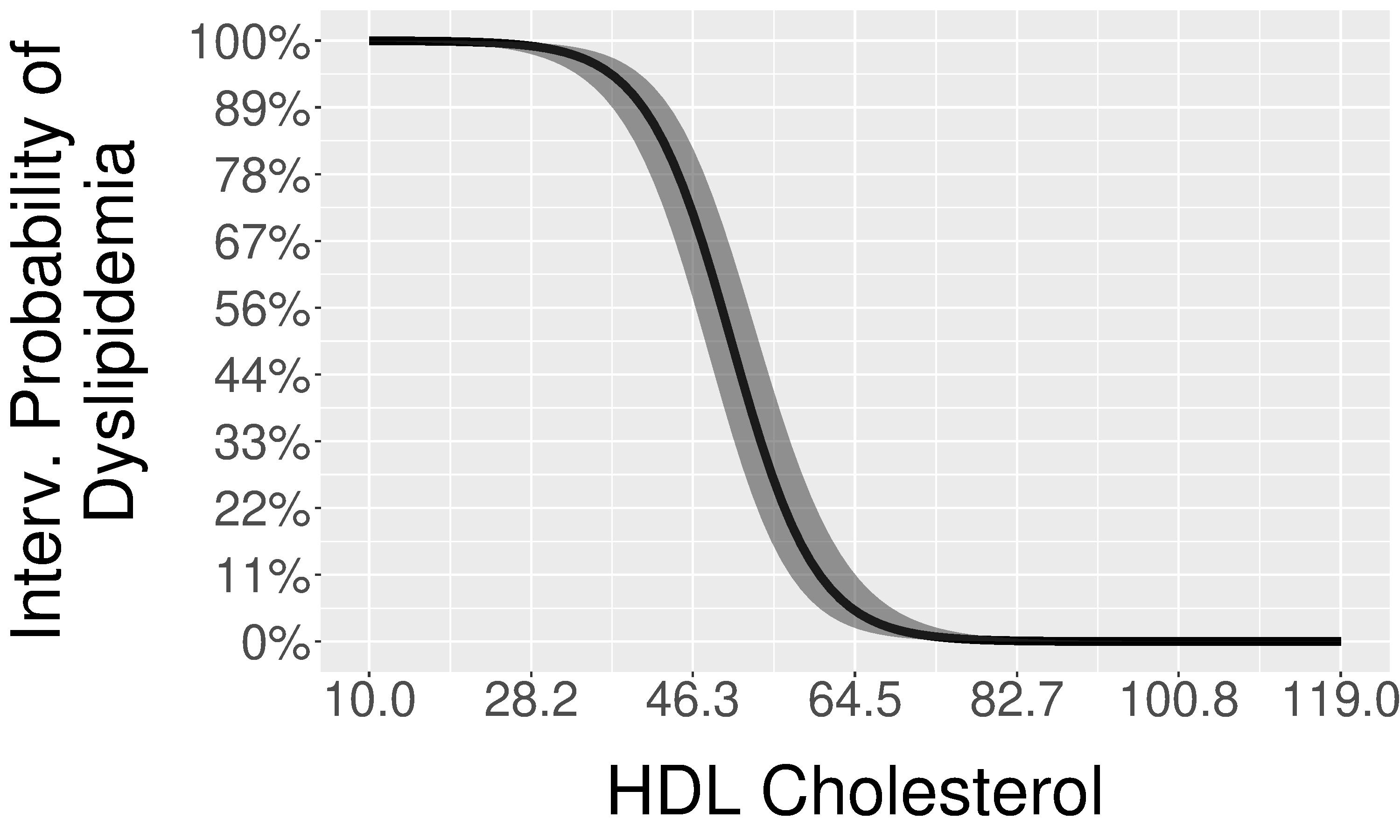

Supplement: Supplementary file 2 [file DataSheet2.zip › figures/Fig4G.jpg]

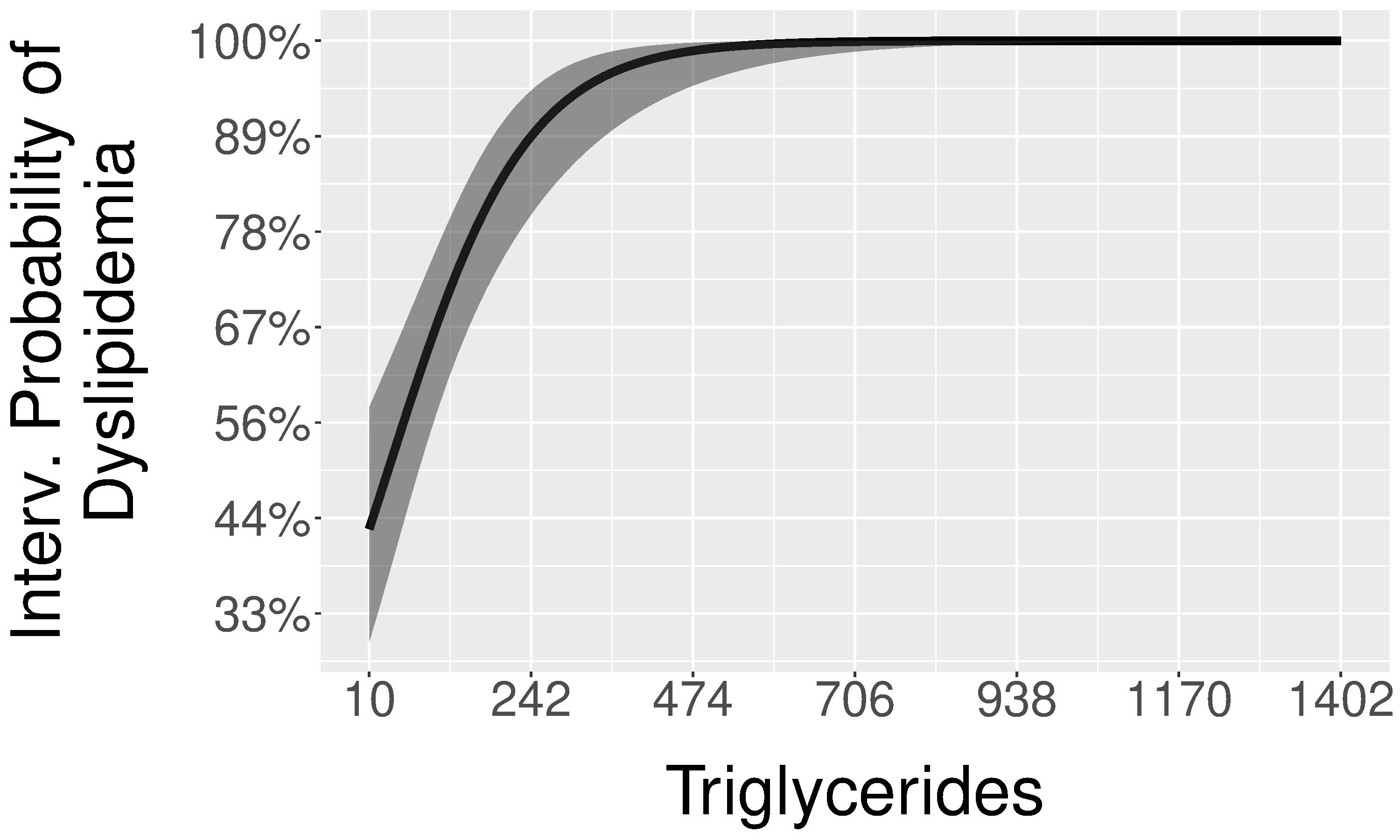

Supplement: Supplementary file 2 [file DataSheet2.zip › figures/Fig4F.jpg]

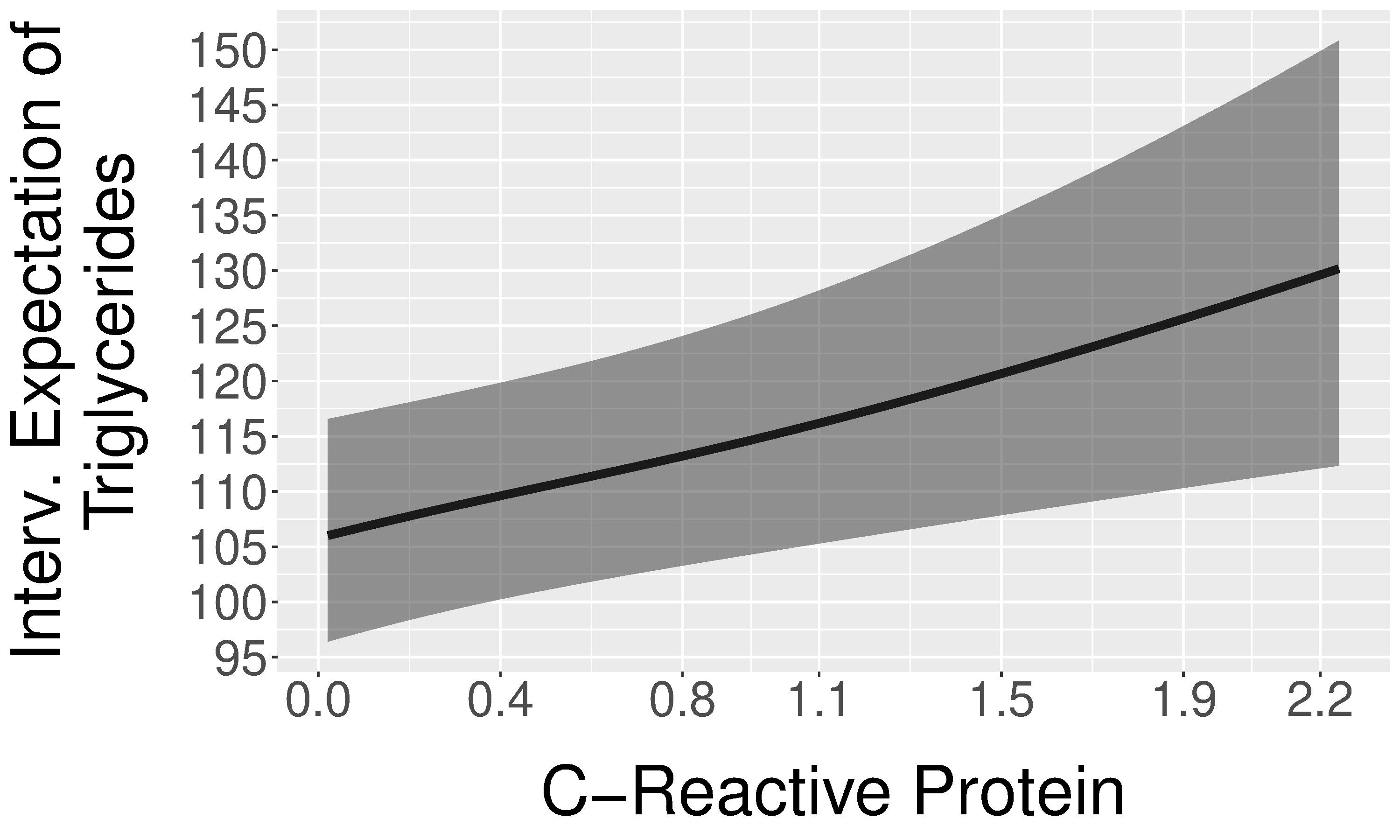

Supplement: Supplementary file 2 [file DataSheet2.zip › figures/Fig4B.jpg]

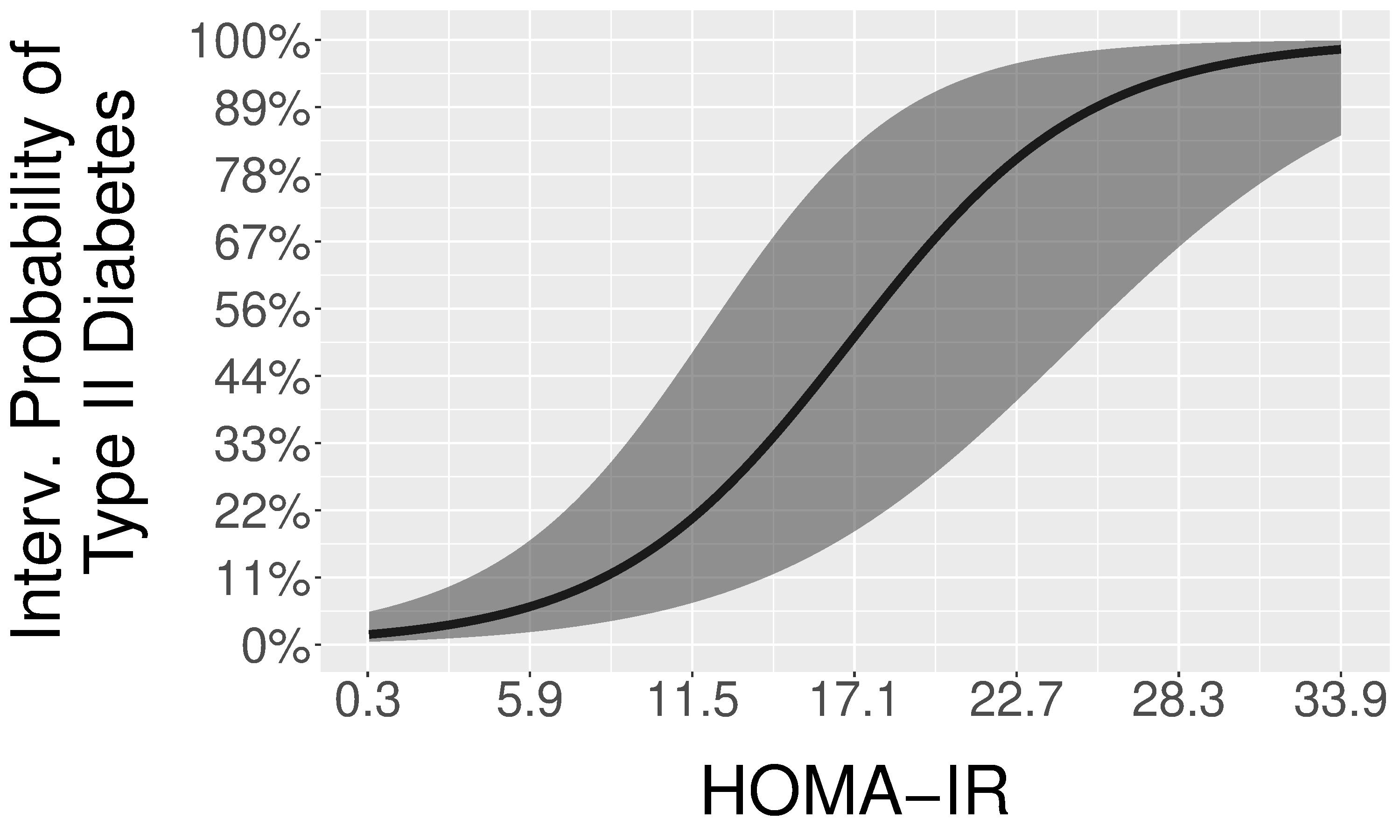

Supplement: Supplementary file 2 [file DataSheet2.zip › figures/Fig4C.jpg]

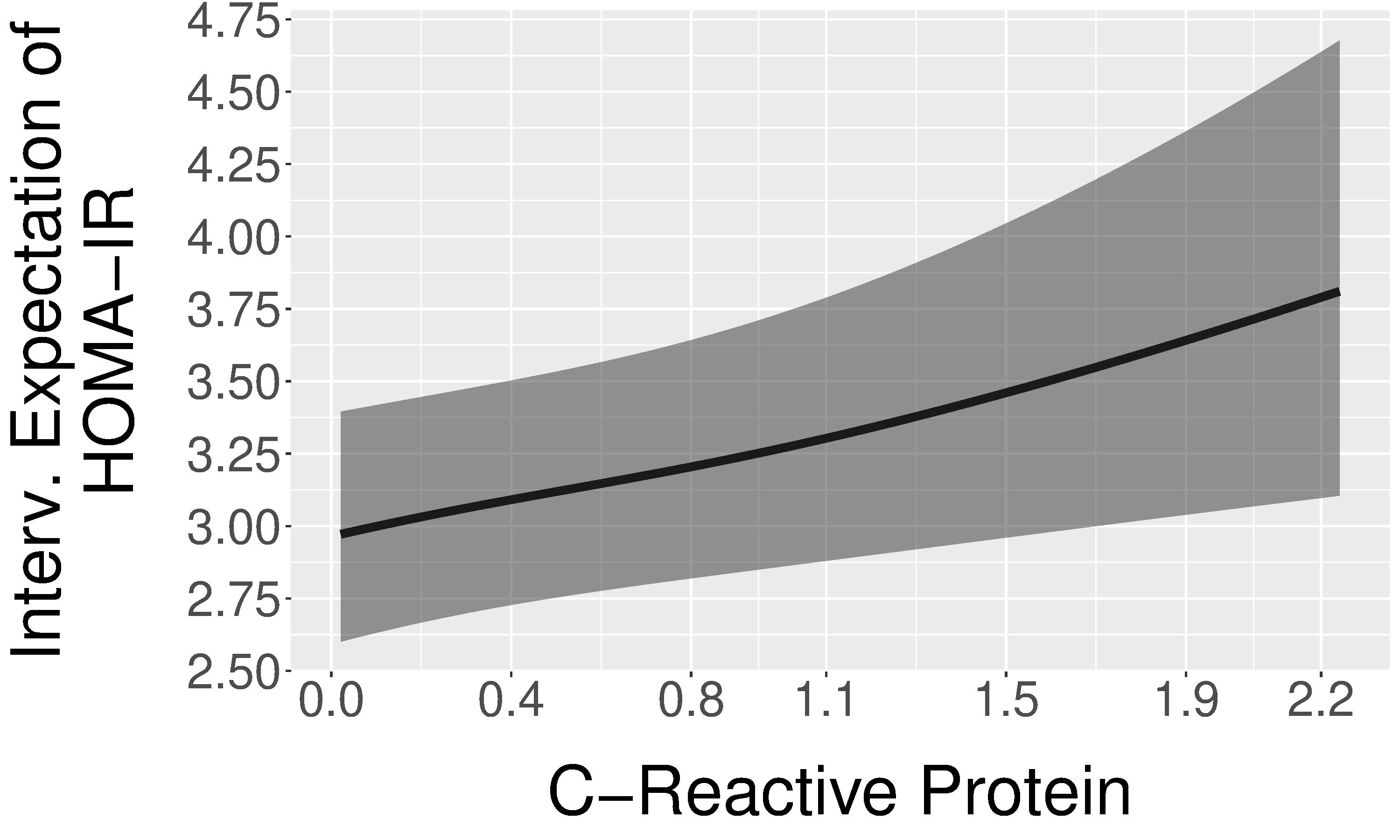

Supplement: Supplementary file 2 [file DataSheet2.zip › figures/Fig4A.jpg]

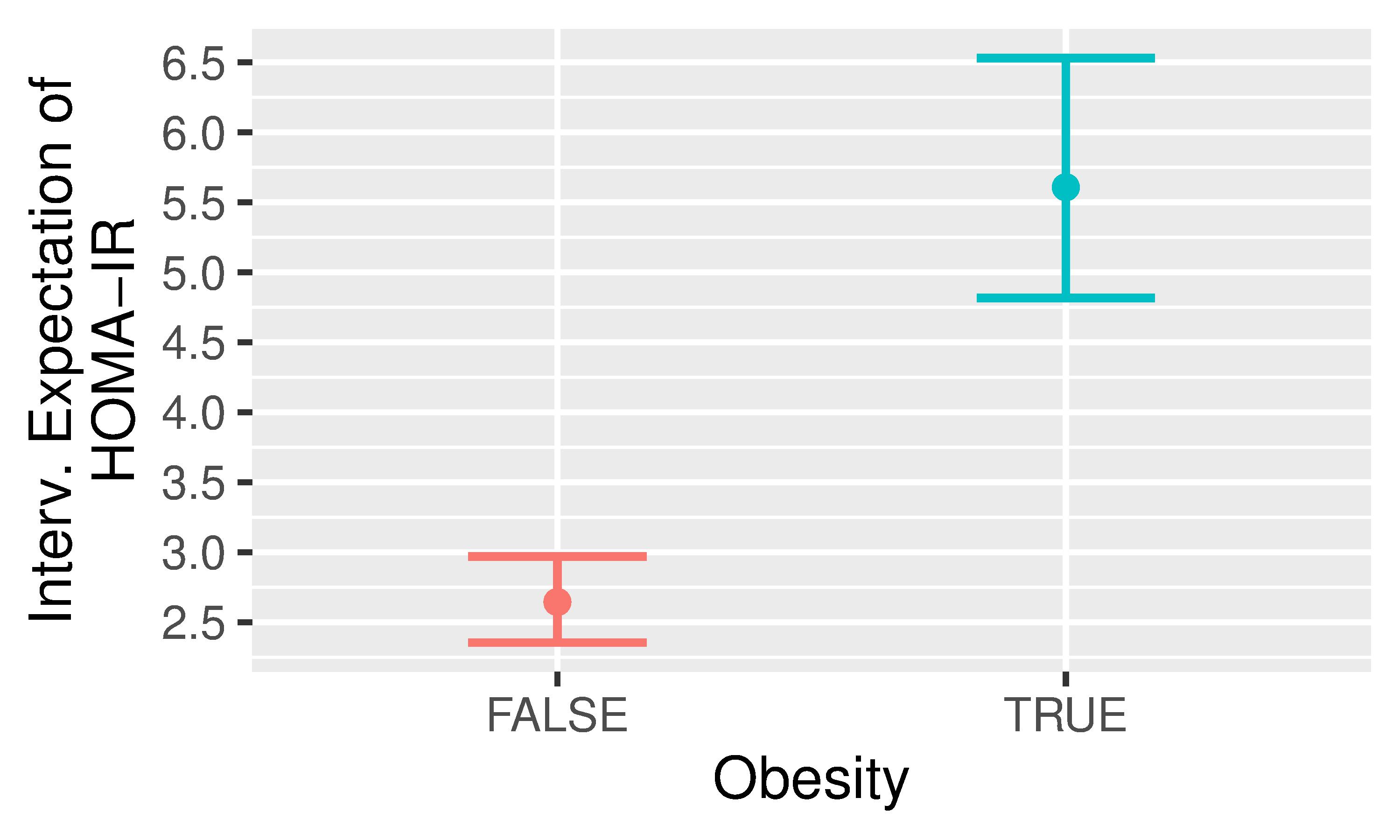

Supplement: Supplementary file 2 [file DataSheet2.zip › figures/FIg3C.jpg]

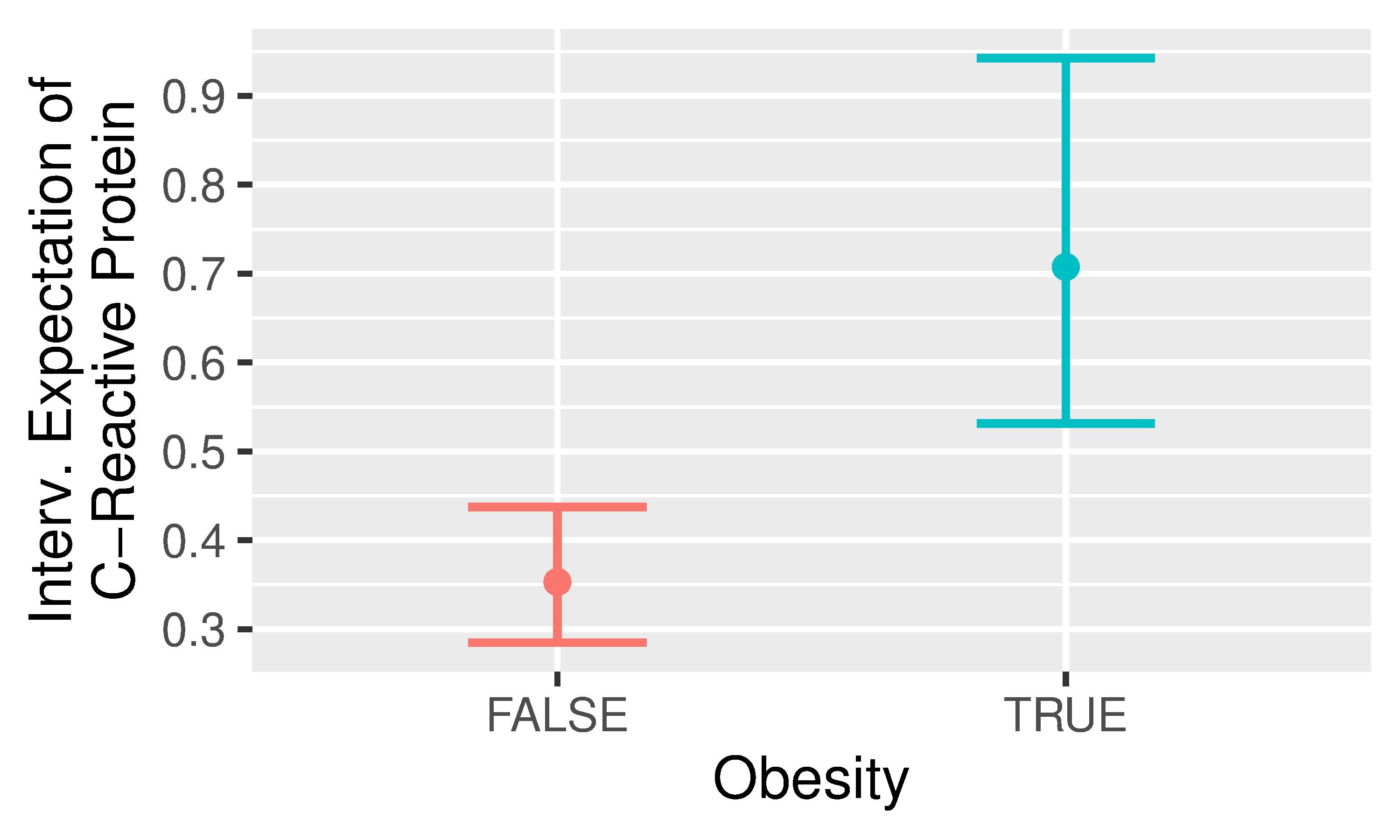

Supplement: Supplementary file 2 [file DataSheet2.zip › figures/FIg3B.jpg]

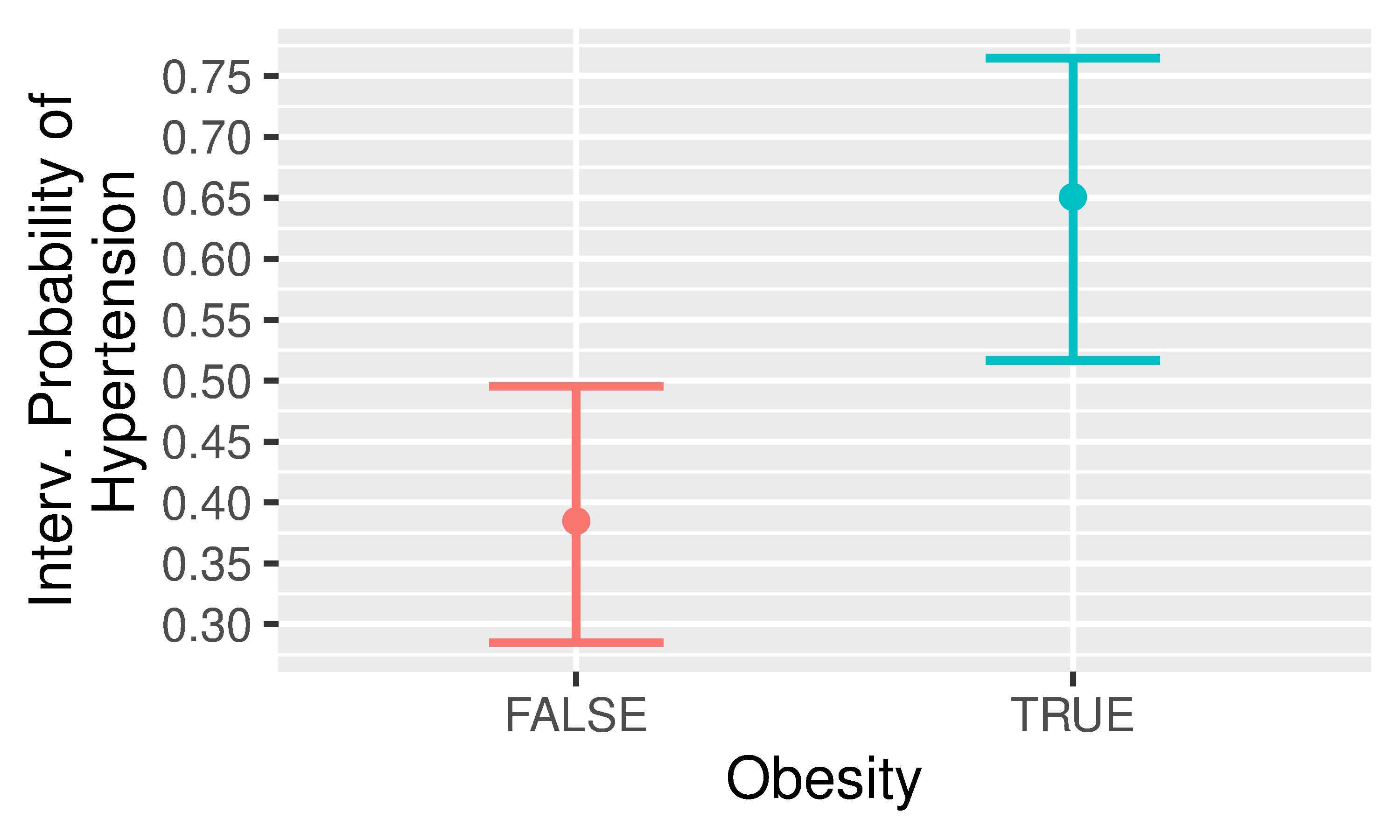

Supplement: Supplementary file 2 [file DataSheet2.zip › figures/Fig3A.jpg]

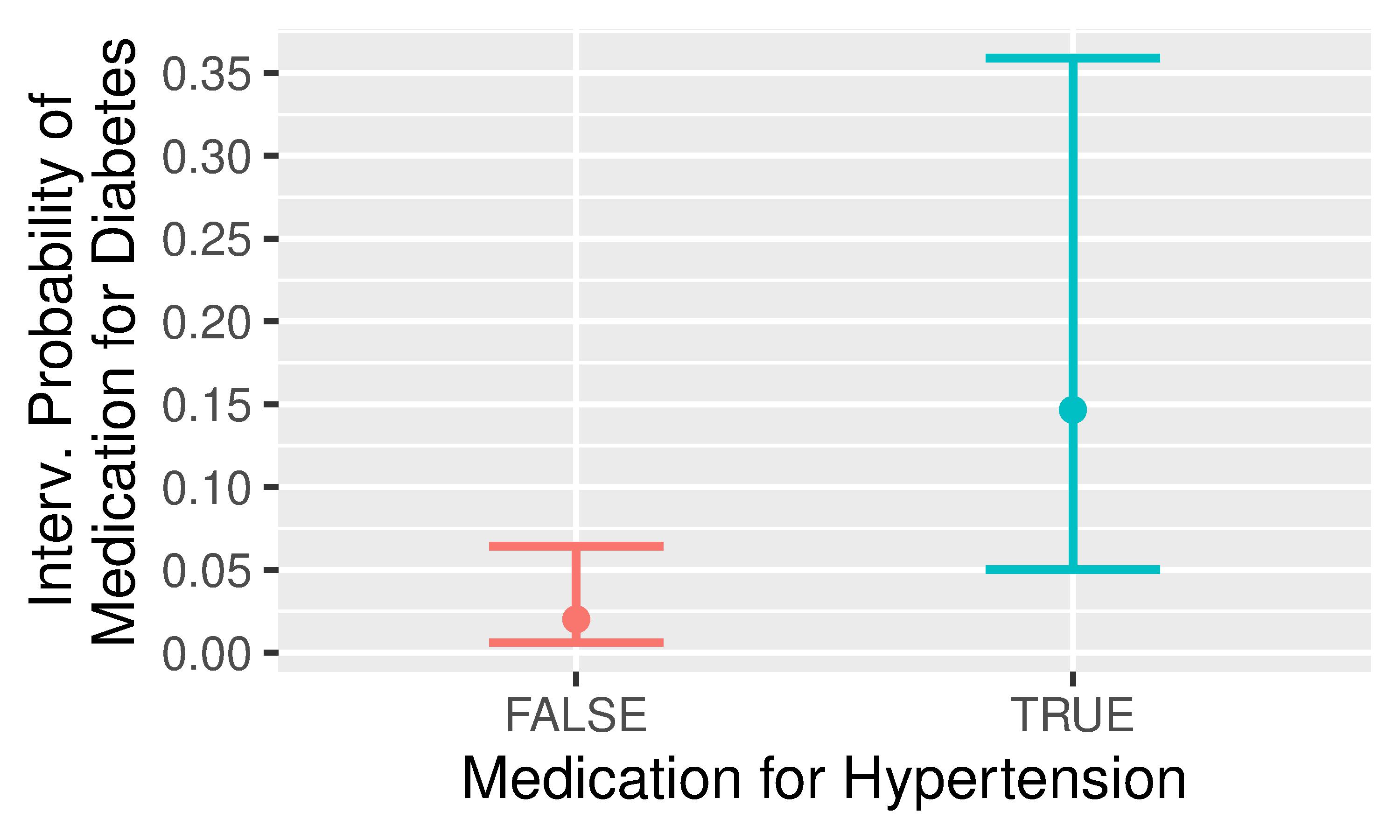

Supplement: Supplementary file 2 [file DataSheet2.zip › figures/Fig3E.jpg]

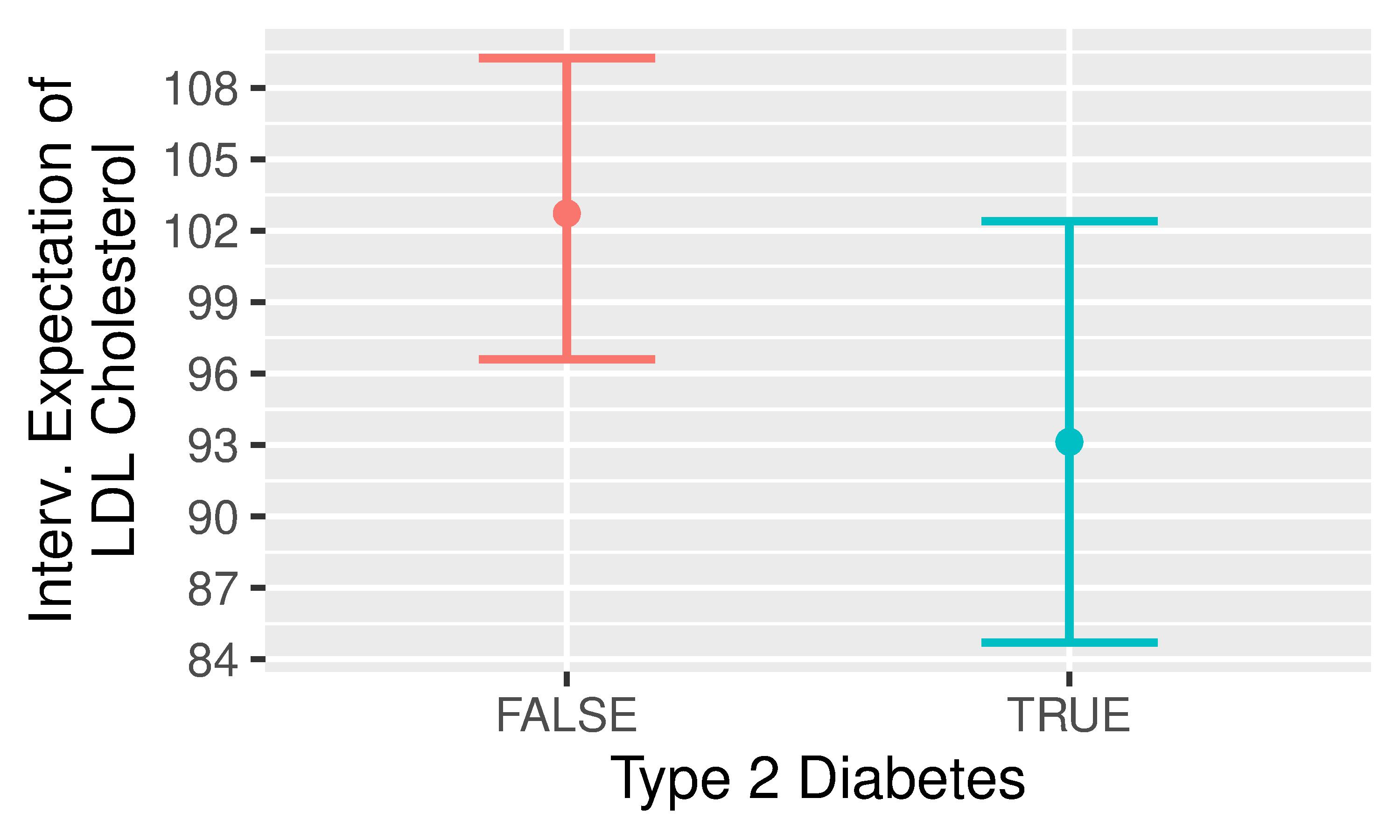

Supplement: Supplementary file 2 [file DataSheet2.zip › figures/FIg3D.jpg]
